# Transcriptional profiling of sugarcane leaves and roots under progressive osmotic stress reveals a regulated coordination of gene expression in a spatiotemporal manner

**DOI:** 10.1371/journal.pone.0189271

**Published:** 2017-12-11

**Authors:** Alejandro Pereira-Santana, Edyciel J. Alvarado-Robledo, Jesus A. Zamora-Briseño, Jorge T. Ayala-Sumuano, Victor M. Gonzalez-Mendoza, Francisco Espadas-Gil, Luis D. Alcaraz, Enrique Castaño, Miguel A. Keb-Llanes, Felipe Sanchez-Teyer, Luis Carlos Rodriguez-Zapata

**Affiliations:** 1 Unidad de Biotecnología, Centro de Investigación Científica de Yucatán, Mérida, Yucatán, México; 2 Departamento de Neurobiología del Desarrollo y Neurofisiología, Instituto de Neurobiología, Universidad Nacional Autónoma de México, Campus Juriquilla, Querétaro, México; 3 Unidad de Bioquímica y Biología Molecular de Plantas, Centro de Investigación Científica de Yucatán, Mérida, Yucatán, México; 4 Laboratorio Nacional de Ciencias de la Sostenibilidad (LANCIS), Instituto de Ecología, Universidad Nacional Autónoma de México, Cd. Mx, México; Institute of Genetics and Developmental Biology Chinese Academy of Sciences, CHINA

## Abstract

Sugarcane is one of the most important crops worldwide and is a key plant for the global production of sucrose. Sugarcane cultivation is severely affected by drought stress and it is considered as the major limiting factor for their productivity. In recent years, this plant has been subjected to intensive research focused on improving its resilience against water scarcity; particularly the molecular mechanisms in response to drought stress have become an underlying issue for its improvement. To better understand water stress and the molecular mechanisms we performed a *de novo* transcriptomic assembly of sugarcane (var. Mex 69–290). A total of 16 libraries were sequenced in a 2x100 bp configuration on a HiSeq-Illumina platform. A total of 536 and 750 genes were differentially up-regulated along with the stress treatments for leave and root tissues respectively, while 1093 and 531 genes were differentially down-regulated in leaves and roots respectively. Gene Ontology functional analysis showed that genes related to response of water deprivation, heat, abscisic acid, and flavonoid biosynthesis were enriched during stress treatment in our study. The reliability of the observed expression patterns was confirmed by RT-qPCR. Additionally, several physiological parameters of sugarcane were significantly affected due to stress imposition. The results of this study may help identify useful target genes and provide tissue-specific data set of genes that are differentially expressed in response to osmotic stress, as well as a complete analysis of the main groups is significantly enriched under this condition. This study provides a useful benchmark for improving drought tolerance in sugarcane and other economically important grass species.

## Introduction

*Saccharum officinarum* L. (Sugarcane) belongs to the Poaceae family, an economically important group of tropical and subtropical grasses, comprising plants such as maize, wheat, rice, and sorghum. Sugarcane is a key plant for the global production of sucrose; as well to the bioethanol industry, animal feed and molasses [1, 2]. Moreover, sugarcane is one of the most important productive crops around the world, yielding an average of 1.66 billion of tons per year [3].

Sugarcane cultivation is severely affected by drought stress, and this is the major limiting factor on its productivity worldwide [4]. The global climate change projections in the last years points to a bleak scenario where drought is the major problem for sugarcane crop production [5, 6]. Drought has affected large agricultural areas and rural communities, resulting in imbalance at micro and macroeconomics levels [7]. Even more, drought projections point to a 25% decrease in production by 2080 [8, 9].

In recent years, sugarcane has been subjected to intensive research focused on improving its resilience against water scarcity; particularly the molecular mechanisms in response to drought stress have become an underlying issue for its improvement. Relatively few drought-responsive genes have been described so far, and these studies have focused only on leaves [10–13]. Thereby, novel methodologies and techniques have been useful for the identification of genes that respond to drought stress and they have been used to improve stress tolerance and raise crop productivity [14]. RNA-Seq can analyse species without a sequenced genome on a genomic scale and can provide useful transcriptional data for a deeper understanding of molecular processes governing the plant response to a specific stress [15, 16]. Nowadays, transcriptomic approaches on sugarcane by using RNA-seq have been centred on sugar accumulation in the cane and leaves development [17, 18].

In the present study, we characterized the global transcriptional changes in leaves and roots of sugarcane var. Mex 69–290 under osmotic stress. *De novo* transcriptome analysis was conducted by using plantlets subjected to osmotic stress conditions induced by PEG 8000 treatment. The sugarcane transcriptomes were analysed on leaves and roots after 24, 48, and 72 h of stress induction, and using a non-stressed group as a treatment control. This work represents the most detailed transcriptomic description of sugarcane under water stress condition, up-to-date. We are providing a comprehensive catalog of up and down-regulated genes for leaves and roots, which could be a useful resource for future genetic plant breeding programs in sugarcane as well as other economically important grass species.

## Results

### Physiological characterization of sugarcane plantlets under osmotic stress

**Fig 1:**
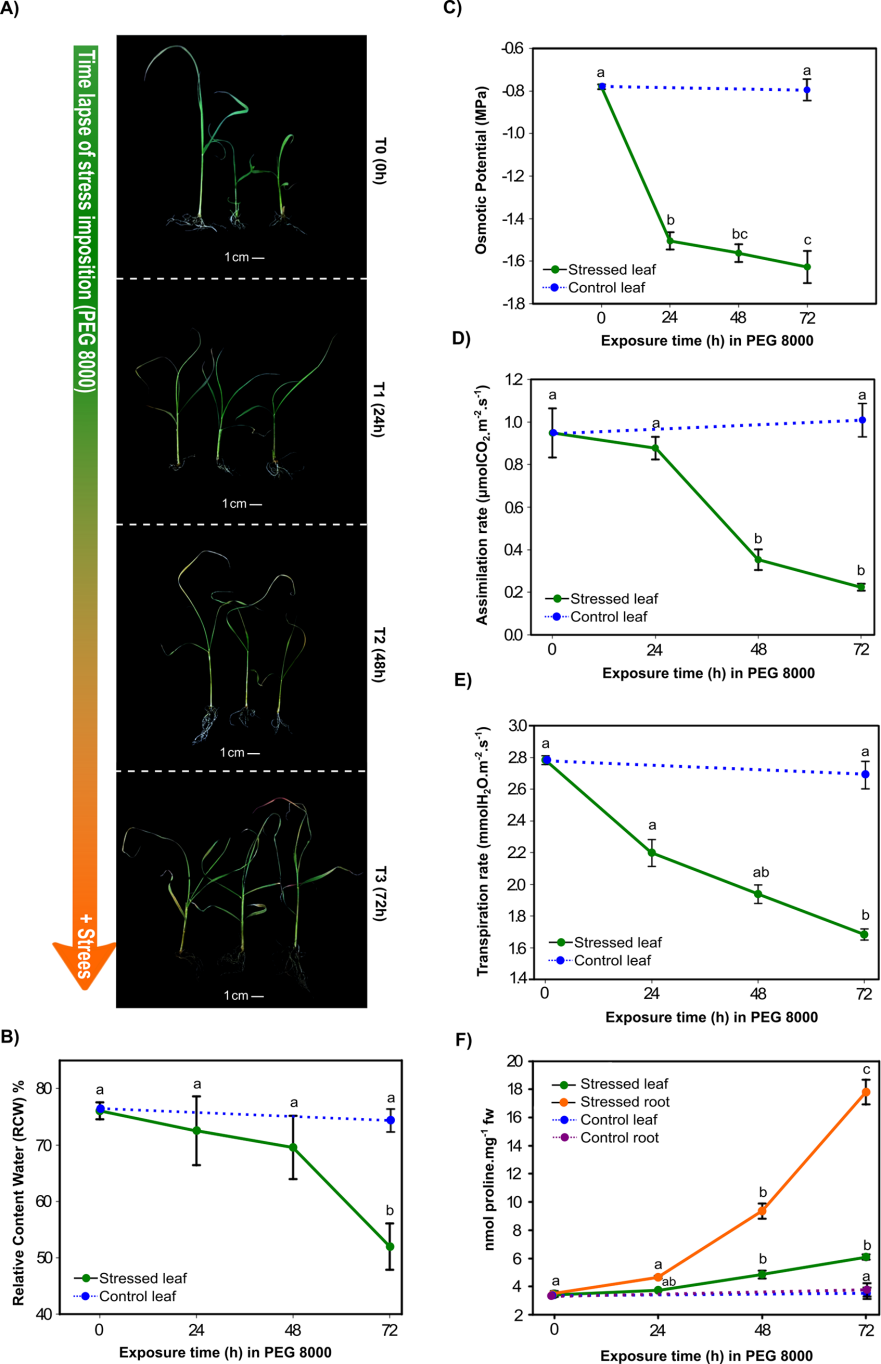
Physiological and biochemical characterization of *in vitro* sugarcane plantlets under osmotic stress induced by PEG 8000. Four physiological parameters and one biochemical parameter were measured in sugarcane plantlets to characterize the effect of the osmotic stress imposition. A) Plantlets exposed to osmotic stress by PEG 8000 (40%). The time of stress exposure (from top to bottom) is indicated by an arrow showing the severity of the stress treatment increased according to time (0, 24, 48, and 72 hours respectively). Physiological measurement of B) Relative water content, C) Osmotic potential, D) Assimilation rate, E) Transpiration rate, and F) Biochemical measurement of proline content in roots and leaves of sugarcane.

Morphological differences were observed between T0 and T1, differences in treatments T2 and T3 included: curled leaves with a generalized loss of turgor and became chlorotic with some degree of apical dead tissue. Additionally, in T3 the root system showed some degree of apparent necrosis (Fig 1A).

These morphological observations are correlated with the physiological measured parameters. For T3 treatment a statically difference in RWC was found (Tuckey’s HSD, p<0.05) in comparison to T0, T1, and T2, with a reduction of 31.17% with respect to T0, indicating a loss of the cell turgor (Fig 1B). The osmotic potential showed a reduction of approximately 60% in T3 in relation to the control (Fig 1C). Statistical differences in the CO_2_ assimilation were found in T2 and T3, with reductions of 62.7% and 76%, respectively (Fig 1D). Transpiration rate was significantly different in T2 and T3, with reductions of 30% and 39%, respectively (Fig 1E). Proline content was tested and showed increased in both leaves and roots during treatment (Fig 1F). Leaves showed the biggest increment in proline during T3 treatment, increasing from 3.5 to 6 nmol mg^-1^ FW in T3. Higher accumulation of proline was detected in roots with 3 and 6 fold increments for T2 and T3, respectively.

### Transcriptome analysis

The sugarcane transcriptome consisted in 252,702 transcripts (all assembled sequences containing isoforms) and 140,339 unigenes (the longer fragments without N bases). The summary of our transcriptome is shown in Table 1. After *de novo* assembly each unigene was compared by BLASTX (E-value 1e^-20^) against several databases. A total of 31,130 unigenes (22.18%) were annotated against the PlantRef protein database from NCBI.

**Table 1:** Summary of transcriptome data for sugarcane var. Mex 69–290.

	Number	Percentage (%)
Raw reads	412,440,102	
Clean reads (Q>30)	410,382,788	
Average of GC% content	48.18	
Total aligned reads	361,376,210	
Proper aligned pairs	243,487,632	67.38
Improper aligned pairs	86,259,650	23.87
Left only	17,107,872	4.73
Right only	14,521,056	4.02
Total transcripts	252,702	
N50 length (Transcripts)	1,430	
Total unigenes	140,339	
N50 length (Unigenes)	1,213	
Unigenes (300–1,000 nt)	63,273	45.08
Unigenes (1,001–3,000 nt)	20,673	14.73
Unigenes (3,001–10,000)	2,463	1.75
Unigenes (10,001 ≥)	16	0.01
Unigenes in Swiss-Prot database	19,211	13.68
Unigenes in TrEMBL database	38,798	27.63
Unigenes in Pfam database	29,212	20.8
Unigenes in CDD database	25,814	18.38
Unigenes in Plant ref-seq database	31,130	22.18
Unigenes in GO database	19,372	13.79

**Fig 2:**
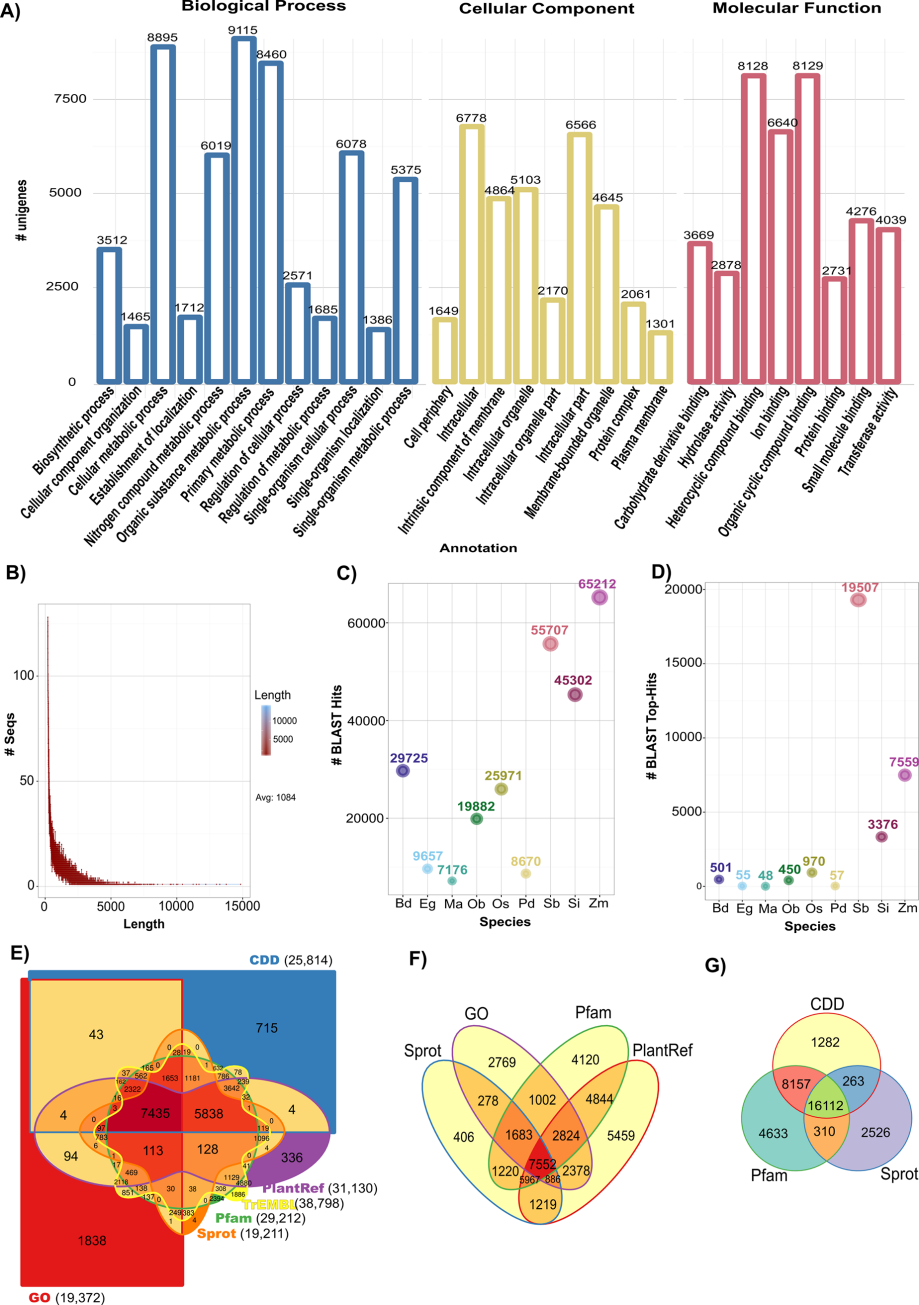
Summary of the annotated unigenes of *S*. *officinarum* var. Mex 69–290 transcriptome. A) Gene Ontology (GO) classification of the unigenes annotated. B) Distribution lengths of the assembled unigenes. C) Distribution of BLASTX-hits for annotated unigenes among the 10 principal plant species represented in the PlantRef protein database. D) Distribution of the closest homology matches (Top BLASTX-hits) for mapped unigenes in the ten principal species represented in the PlantRef protein database. E) Venn diagram is showing BLAST results of unique and shared unigenes among CDD, GO, Pfam, Sprot, TrEMBL, and PlantRef databases. F) Venn diagram showing shared unigenes among GO, Pfam, Sprot, and PlantRef databases. G) Venn diagram showing the BLAST results for the three principal protein databases, Pfam, Sprot, and CDD. Plant species names were coded for C) and D) as follows: Bd = *Brachypodium distachyon*, Eg = *Elaeis guineensis*, Ma = *Musa acuminata* subsp. Malaccensis, Ob = *Oryza brachyantha*, Os = *Oryza sativa* Japonica, Pd = *Phoenix dactylifera*, Sb = *Sorghum bicolor*, Si = *Setaria italica*, and Zm = *Zea mays*.

All unigenes were mapped against the GO terms database (S1 File). A total of 19,372 unigenes were classified in 29 subcategories belonging to three major GO categories: Biological Process, Molecular Function, and Cellular Component (Fig 2A). Among the Biological Process category, “Organic substance metabolic process (16.1%)”, “Cellular metabolic process (15.8%)”, and “Primary metabolic process (15%)” were the main functional groups, followed by “Single-organism cellular process (10.8%)” and “Nitrogen compound metabolic process (10.6%)”. For the Cellular Component, “Intracellular (37.9%)” and “Membrane-bounded organelle (13.2%)” were the most abundant subcategory in this group, “Organic cycling compound binding (20%)” and “Heterocyclic compound binding (20%)” were the two main groups into the Molecular Function category.

The length of unigenes ranged from 201 bp to 14,847 bp, with an average of 1,084 bp and a N50 length of 1,213 bp (N50 means that 50% of the entire assembly is contained in Transcripts equal to or larger than this value; Fig 2B). Among the annotated unigenes, BLAST result had homology to *Zea mays*, *Sorghum bicolor*, *and Setaria italica* grass species (Fig 2C), higher identity of the matches was against *S*. *bicolor* (Fig 2D). All unigenes were annotated by mapping them to several public databases showed in Table 1, and results were depicted by using Venn diagrams (Fig 2E) and also, different Venn diagrams were plotted for the easiest comparison among databases (Fig 2F and 2G).

**Fig 3:**
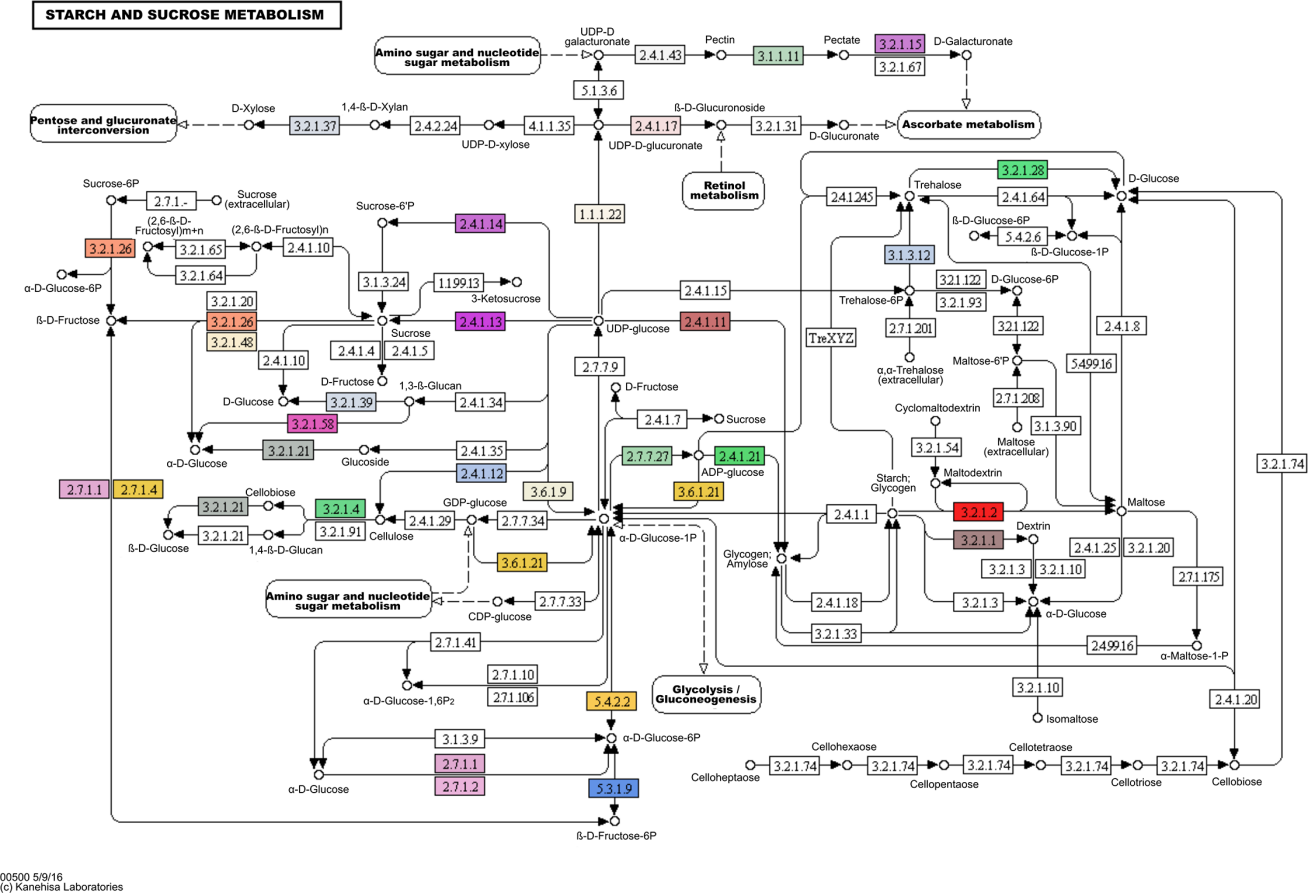
Unigenes involved in the starch and sucrose metabolism pathway according to KEGG database. Predicted coded enzymes for starch and sucrose metabolism are marked as colored boxes (S2 File). Colors are used simply to differentiate each enzyme (one color for each EC). Reprinted from Kanehisa Laboratories under a CC BY license, with permission from Miwako Matsumoto, original copyright 5/9/16 (S3 File).

KEGG was used to predict metabolic functions of the unigenes [19]. Altogether, 6,840 unigenes were assigned to 129 pathways, including “Photosynthesis”, “Nitrogen metabolism”, “Carbon fixation in photosynthetic organisms”, “Glycolysis/Gluconeogenesis”, “Purine metabolism”, “Glutathione metabolism”, among others (S2 File). The unigenes identified for the “Starch and sucrose metabolism” pathway are shown in Fig 3.

### Sample clustering analysis for sugarcane libraries

We used principal component analysis (PCA), gene expression patterns during osmotic stress, and hierarchical clustering analysis (HCA) to obtain time- and tissue-specific gene profiling. PCA shows similarity among the non-stressed samples for both leaf and root tissues and was clustered as a neighbouring group, showing a similar transcript level in comparison to stressed samples.

**Fig 4:**
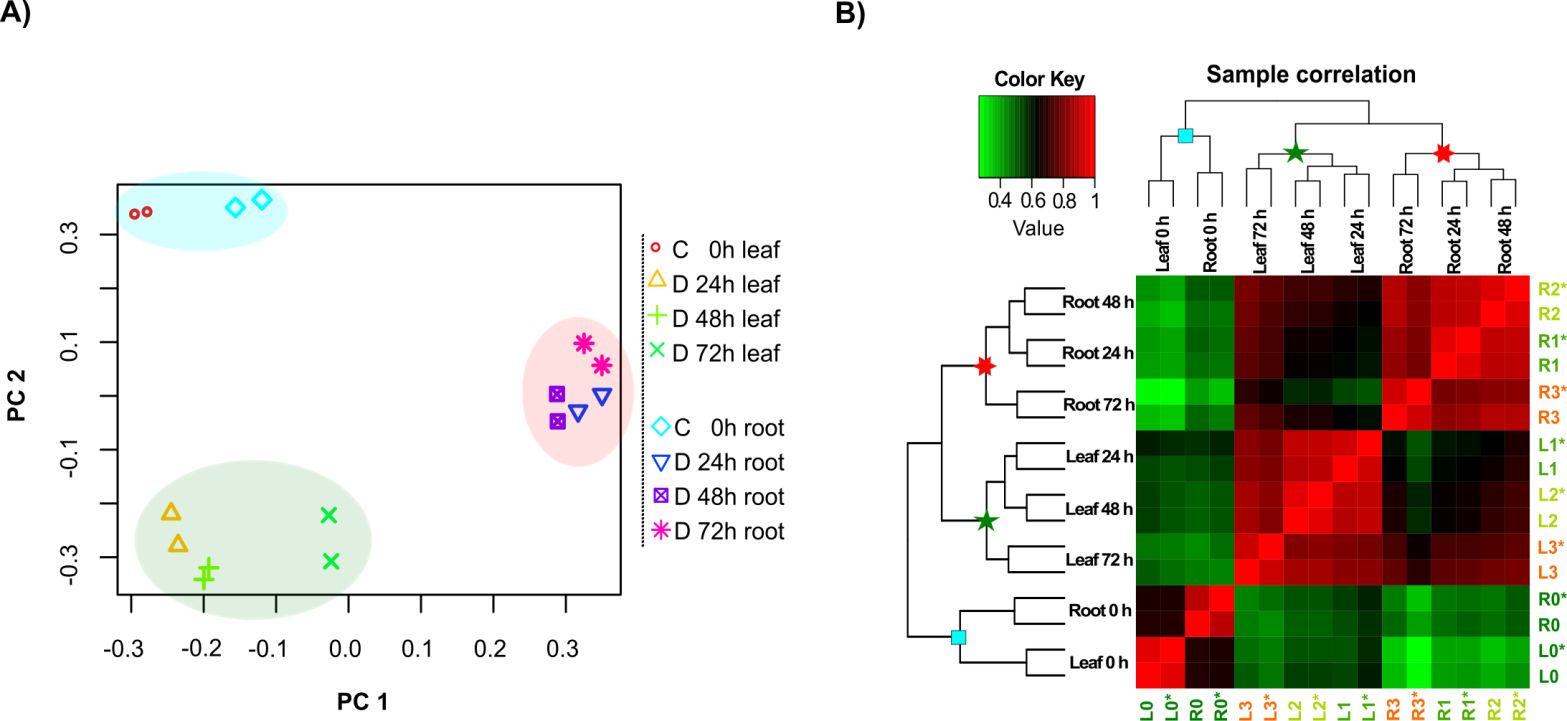
Correlation of sample replicates and hierarchical clustering analysis of gene expression levels in each sample (0, 24, 48, and 72 h) under osmotic stress treatment. A) Sample relatedness is plotted using the first two principal component (PCs) showing the variability between replicates and different treatments. Non-stressed leaf and root samples are in blue shade background. In dark-green and in red background colors are the samples corresponding to stressed leaves and roots (24, 48, 72 h ASI), respectively. C = control, D = Drought. B) Correlation dendrogram of gene expression in all samples. Correlation *r* values are coded into key colors where green indicates lower *r* values; reds indicates higher *r* above the mean. Blue squares in the nodes represent non-stressed samples, green stars represent leaf stressed samples, and the red stars represent root stressed samples. L = leaf, R = root, L0 and R0 = non-stress treatment, L1 and R1 = 24 h treatment ASI, L2 and R2 = 48 h treatment ASI, L3 and R3 = 72 h treatment ASI. * = biological replicate.

The stressed leaf samples (24, 48, and 72 h after stress induction (ASI)) were grouped together as a negative correlated cluster, in comparison to the root stressed samples, that were grouped apart from leaves, as a tissue specific cluster (Fig 4A).

From here on we will refer to the following codes: L = leaf, R = root, L0 and R0 = non-stress treatment, L1 and R1 = 24 h ASI, L2 and R2 = 48 h treatment ASI, L3 and R3 = 72 h treatment ASI. Similar to PCA, the HCA shows that L0 was assigned to the adjacent node of R0, which corresponds to non-stress treatments. L1, L2, and L3 were grouped into the same cluster, corresponding to stress treatment in leaf tissue; while R1, R2, and R3 were grouped in a cluster corresponding to stress treatment in root tissue. This finding demonstrates the gene expression specificity for each tissue during osmotic stress. During the stress treatments, similarities in gene expression pattern were observed between L1, L2, and, L3, and R1, R2, and R3. On the other hand, L0 and R0 were clustered together showing high *r* values above the mean, suggesting a similar pattern in gene expression for a non-stress condition for leaf and root (Fig 4B). The transcriptome data shows that gene expression is significantly altered from the first time of stress imposition (L1 and R1) for both leaf and root tissues of sugarcane sprouts.

### Global analysis of Differentially Expressed Genes (DEGs) during osmotic stress

**Fig 5:**
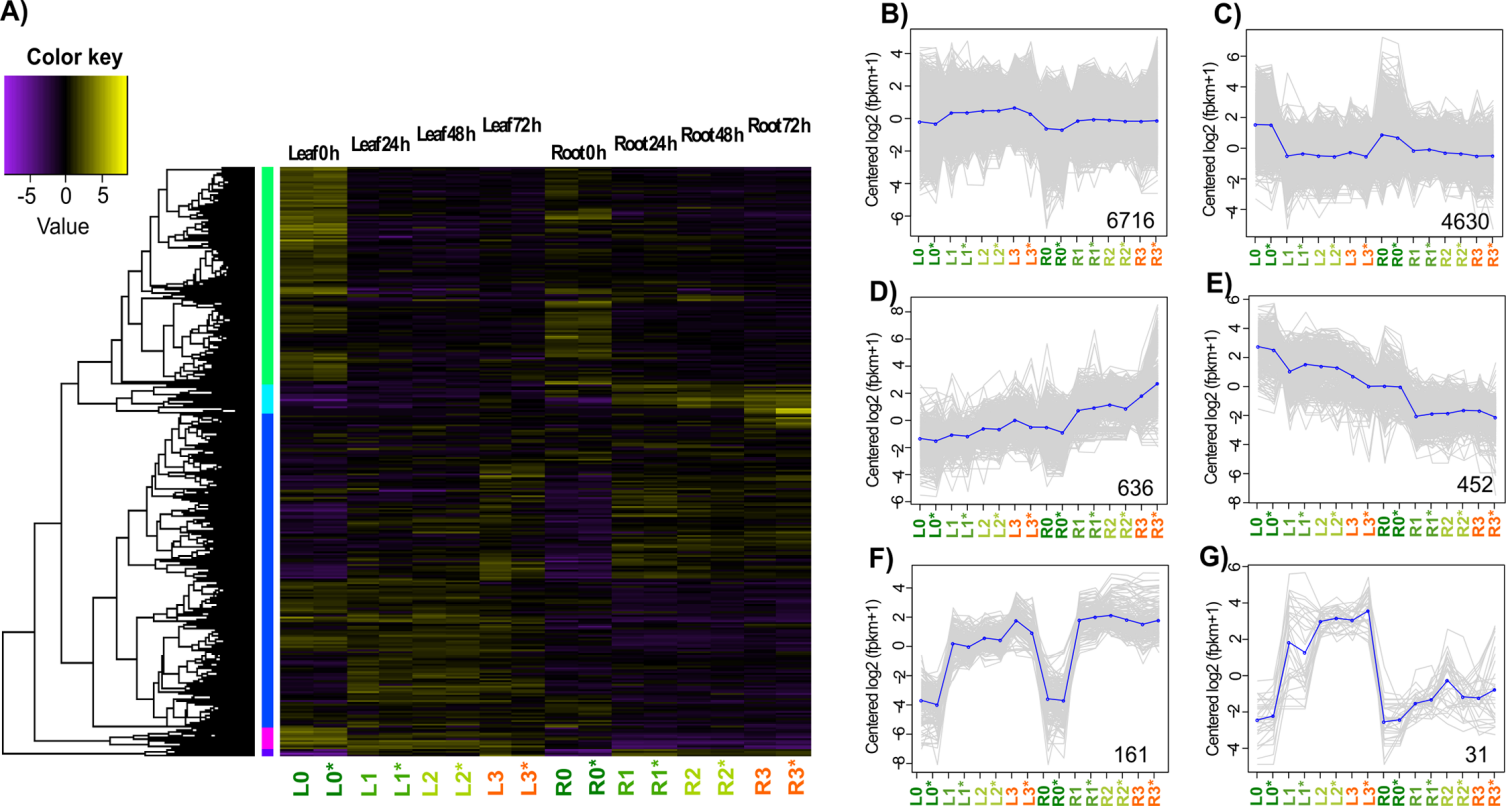
Correlation and clustering analysis of the sugarcane DEGs subjected to osmotic stress treatments. A) Tissue-specific heatmap clustering of 12,662 significant DEGs (FDR ≤ 0.001 and FC ≥2) for the 0, 24, 48, and 72 h treatments ASI. B-G) Osmotic stress DEG profiles for the organ specific behavior clusters. Clusters were built according to their expression patterns during the stress treatments. Gray lines indicate the behavior of each gene into the treatment and the mean expression profile for each cluster is plotted as blue lines. Bottom number indicates the number of time series genes. L = leaf, R = root. L0 and R0 = non-stress treatment, L1 and R1 = 24 h treatment ASI, L2 and R2 = 48 h treatment ASI, L3 and R3 = 72 h treatment ASI. * = biological replicate.

Identification of sugarcane DEGs associated with osmotic stress was done by analysis of gene expression levels and it was quantified by RSEM software v1.2.27 [20], all read counts were normalized to FPKM values. Pearson’s correlation coefficients were higher than 0.95 in all cases (S1 Fig). Dynamic changes of gene expression patterns were retrieved by using a gathering cut-off value of fold change (FC) >2 and an adjusted p-value (FDR) of <0.001. On the basis of similar expression kinetics, 12,662 DEGs were depicted by a heatmap analysis to show major clusters of DEGs according to tissue specificity and time-stress treatment (Fig 5A). Similar expression profiles were observed for T0 samples and specific clusters were denoted for stressed samples (T1, T2, and T3) depending on plant tissues. Temporal expression patterns of genes following osmotic stress treatments are shown in Fig 5A where it is partitioned into six expression clusters (log2-transformed) with similar expression profiles (Fig 5B–5G). Each profile represents a group of genes that exhibited similar expression trend. The six clusters were generated by cutting the hierarchically clustered gene tree (5A) at 60% of the tree height (recommend; see methods). In cluster 5B, where most of the genes were grouped, the mean suggests few significant changes among treatments. In cluster 5C, the 4,630 clustered showed a down-regulation in the stress treatments for both leaf and root tissues. In contrast, cluster 5D and cluster 5E showed an opposite trend, where genes in cluster 5D were down-regulated on stress treatments and genes in cluster 5E were up-regulated on the stress treatments. The most drastical regulation trend was shown in clusters 5F and 5G, where genes were quickly up-regulated by stress treatments. Only genes belonging to root tissues in cluster 5G showed a low increment in the transcriptional responses.

### DEGs in response to osmotic stress treatments in sugarcane

**Fig 6:**
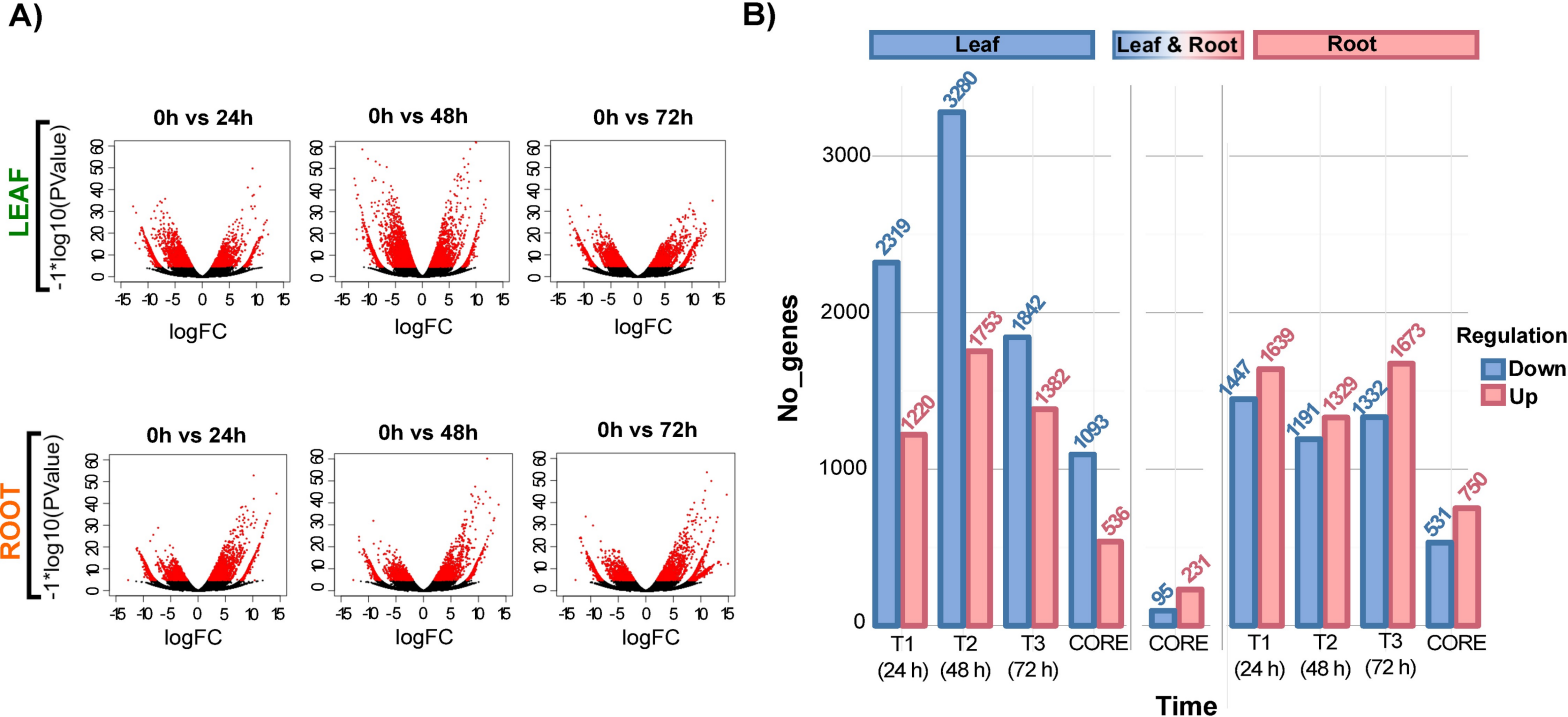
Differentially expressed gene analysis along the osmotic stress treatments in sugarcane. A) Volcano plots show DEGs in each of the stress treatments for leaf and root tissues. The x-axis represents the log2FoldChange of genes, and the y-axis represents the statistical significance degree by–log10 (PValue). DEGs are shown in red dots and were detected using their statistical significance differences (FDR<0.001 and a log2FoldChange>2). B) Comparison of the up- and down-regulated DEGs for leaf and root tissues in all three stress treatments and comparison of shared genes among all the treatments (CORE gene sets).

DEGs of leaves and roots grouped out of all the stress-time series. The volcano plots show that more genes were up- and down-regulated in T2 (48h vs. control) than in T1 (24 h vs. control), or T3 (72 h vs. control) for leaf tissues (Fig 6A). Red dots inside volcano plots show DEG with a Fold Change > 2 and a FDR<0.001. The volcano plots show that large number of genes are differentially expressed across the stress-treatments. According to this, we have that T2>T1>T3 for down-regulated genes and T2>T3>T1 for up-regulated genes in leaf tissues. For root tissues a lower amount of DEGs was observed compared to leaves, the relationship for down-regulated genes was T1>T3>T2 and T3>T1>T2 for up-regulated genes in root tissues. A total of 11,796 genes were differentially regulated in leaves, of which 7,441 were down-regulated, and 4,355 were up-regulated. For root tissues, a total of 8,611 genes were differentially regulated, of which 3,970 were down-regulated, and 4,641 were up-regulated. (Fig 6B). A total of 95 down-regulated genes were shared among leaf and root tissues for all three stress treatments and 231 up-regulated genes were shared for both leaf and root tissues, in all the time-stress treatments.

**Fig 7:**
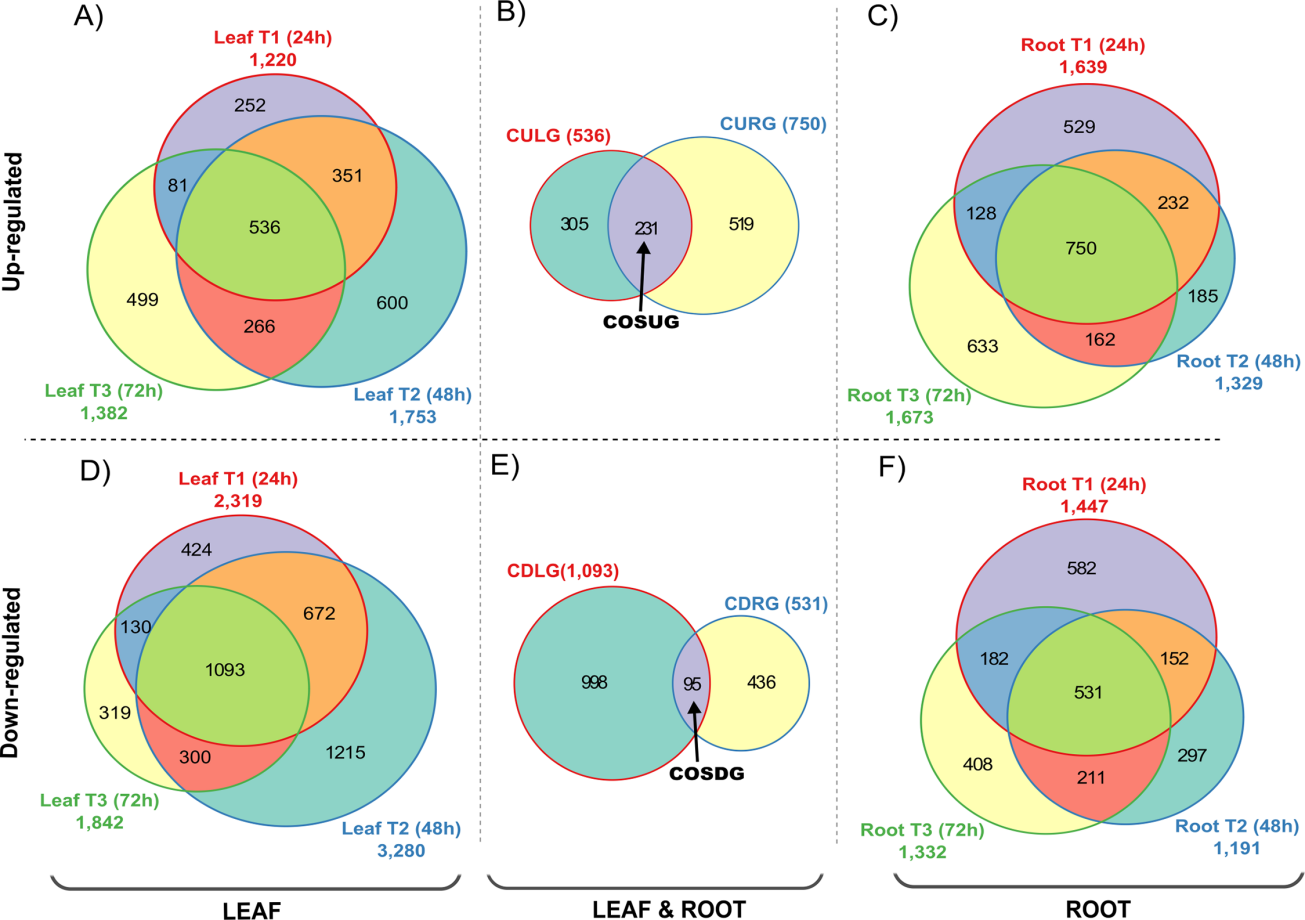
Comparative analysis of the unique and shared DEGs among the osmotic stress treatments for sugarcane var. Mex 69–290. The Venn diagrams show the overlap of the DEGs for leaves and roots submitted to a 3 time-series of osmotic stress treatments. A and C) Comparison among the genes differentially up-regulated for the stress treatments for leaf and root tissues respectively. D and F) Comparison among the genes differentially down-regulated for the stress treatments for leaf and root tissues respectively. B and E) Comparison among the core set of up- and down-regulated genes respectively for both tissues. CULG = CORE of Up-regulated Leaf Genes, CURG = CORE of Up-regulated Root genes, COSUG = CORE of Osmotic Stress Up-regulated Genes, CDLG = CORE of Down-regulated Leaf Genes, CDRG = CORE of Down-regulated Root Genes, and COSDG = CORE of Osmotic Stress Down-regulated Genes. The Venn diagrams were done by using Vennerable R package.

For leaf tissues, 3,539 transcripts were found to be differentially expressed in T1 treatment (24 h ASI), which included 1,220 up-regulated genes (Fig 7A) and 2,319 down-regulated genes (Fig 7D). For T2 treatment (48 h ASI), 5,033 genes were differentially expressed, including 1,753 up-regulated transcripts (Fig 7A) and 3,280 down-regulated transcripts (Fig 7D), and finally, for T3 treatment (72 h ASI), 3,224 transcripts were found differentially expressed, which included 1,382 up-regulated transcripts (Fig 7A) and 1,842 down-regulated transcripts (Fig 7D).

In the case of the up-regulated genes in leaf tissues, 887 genes were shared between T1 and T2, 802 genes were shared between T2 and T3, and 617 genes were shared between T1 and T3. In addition, 536 genes were shared between the three treatments (Fig 7A) and we decided to call it the “CORE of Up-regulated Leaf Genes” (CULG). For the down-regulated genes in leaf tissues, 1,765 genes were shared between T1 and T2, 1,393 genes were shared between T2 and T3, and 1,223 genes were shared between T1 and T3. In the same way, 1,093 genes were shared between the three treatments (Fig 7D) and we decided to call it the “CORE of Down-regulated Leaf Genes” (CDLG).

In roots, the T1 treatment contains 3,086 differentially expressed transcripts, including 1,639 up-regulated genes (Fig 7C) and 1,447 down-regulated genes (Fig 7F). For T2 treatment, 2,520 transcripts were differentially expressed, including 1,329 up regulated (Fig 7C) genes and 1,191 down regulated genes (Fig 7F). Finally, for T3 treatment 3,005 transcripts were differentially regulated, including 1,673 up-regulated genes (Fig 7C) and 1,332 down-regulated genes (Fig 7F).

Among the up-regulated genes in root tissues, 982 genes were shared between T1 and T2, 912 genes were shared between T2 and T3, and 878 genes were shared between T1 and T3 (Fig 7C). Also, 750 genes were shared between the three treatments (Fig 7C) defined as “CORE of Up-regulated Root genes” (CURG). For the down-regulated genes in root tissues, 683 genes were shared between T1 and T2, 742 genes were shared between T2 and T3 treatments and 713 genes were shared between T1 and T3. In the same way, we determined 531 genes shared between the three treatments (Fig 7F) and was defined as the “CORE of Down-regulated Root Genes” (CDRG).

There are 231 genes shared between the COREs of up regulated genes for root and leaf tissues, coded here as “CORE of Osmotic Stress Up-regulated Genes” (COSUG; Fig 7B). On the other hand, we found 95 genes shared between the COREs of down regulated groups for both leaf and root tissues, and was defined as the “CORE of Osmotic Stress Down-regulated Genes” (COSDG; Fig 7E).

There were 1,956 assembled unigenes corresponding to transcription factors (TF), of which 148 were regulated in T1, 174 TF were regulated in T2, and 127 TF were regulated in T3 treatment for leaf tissues (Table 2). In root tissues, 80, 70, and 84 TF were regulated in T1, T2, and T3, respectively. For the up-regulated TFs in leaves, 15 of them were shared among treatments (CULG) and 62 down-regulated TFs were shared among treatments (CDLG). In roots, 19 up-regulated TFs were shared among treatments (CURG) and only 15 down-regulated TFs were shared in the three treatments (CDRG). Interestingly, only two TFs were shared among all the up-regulated genes in leaf and root tissues (COSUG), the gene NAC25-lke and an HSF-C2a. For the down-regulated genes, only two TFs were shared among all the stress treatments for tissues (COSDG), an ERF27-like and a DREB1G-like.

**Table 2:** Stress-related DEGs detected in the CORE sets (CULG and CDLG) of regulated genes in leaves during the osmotic stress treatments.

Gene code	Annotation	Gene expression in Leaf (Log2FC) T1 T2 T3		
DN44309_c2_g1_i1	abscisic acid 8-hydroxylase 3	4.454	4.231	4.414
DN50090_c2_g1_i1	argonaute 2	3.875	2.803	4.233
DN52807_c2_g3_i5	auxin-responsive SAUR36	7.923	8.711	7.496
DN63593_c0_g1_i1	bidirectional sugar transporter SWEET6a	2.97	3.519	4.497
DN58049_c5_g1_i2	Bowman-Birk type trypsin inhibitor	4.403	5.208	5.265
DN47861_c2_g1_i2	bZIP transcription factor superfamily	5.897	5.57	5.528
DN51416_c0_g2_i3	cationic peroxidase SPC4	2.989	3.342	4.076
DN50208_c0_g1_i1	chalcone synthase 1	4.634	6.367	6.809
DN59329_c2_g1_i1	delta-1-pyrroline-5-carboxylate synthase	2.458	2.363	2.466
DN49984_c2_g1_i1	glutathione transferase GST 23	2.318	2.932	3.26
DN55043_c4_g1_i6	heat stress transcription factor C-2a	5.644	5.683	5.969
DN36334_c1_g1_i1	isoflavone 2-hydroxylase	3.547	3.313	4.621
DN60764_c0_g1_i3	laccase-25-like	8.156	9.08	11.625
DN44556_c0_g1_i1	late embryogenesis abundant Lea14-A	8.041	8.475	10.576
DN58475_c3_g1_i9	NAC domain-containing 92	2.586	3.097	4.013
DN49045_c1_g1_i1	NAC transcription factor 25	2.956	3.414	4.697
DN59107_c4_g1_i2	NAC transcription factor 29	3.57	3.557	3.656
DN63909_c11_g1_i1	PHR1-LIKE 1	4.913	4.499	4.739
DN44185_c1_g1_i2	probable phosphatase 2C 59	6.555	6.697	8.807
DN59867_c3_g2_i2	probable proline transporter 2	2.686	2.545	3.197
DN60883_c0_g1_i10	senescence-inducible chloroplast stay-green 1	3.663	4.52	4.54
DN57007_c9_g1_i5	transcription factor MYB1R1	4.622	3.891	4.054
DN47475_c1_g2_i1	zinc finger ZAT12	4.669	5.533	5.619
DN60135_c2_g1_i1	α,α-trehalose-phosphate synthase [UDP-forming] 9	3.631	3.225	3.633
DN38785_c0_g1_i1	ethylene-responsive transcription factor ERF014	-5.442	-6.574	-9.182
DN64637_c1_g1_i1	cationic amino acid transporter 7, chloroplastic	-3.182	-3.221	-2.64
DN52821_c3_g1_i1	cytokinin dehydrogenase 5	-4.087	-4.195	-2.6
DN50424_c7_g1_i1	dehydration-responsive element-binding 1G	-6.884	-9.205	-8.479
DN64458_c0_g1_i1	DNA (cytosine-5)-methyltransferase 1	-4.475	-7.076	-5.266
DN59768_c1_g2_i7	DNA replication licensing factor MCM5	-3.606	-4.513	-5.023
DN43267_c1_g1_i2	ethylene-responsive element binding 2	-9.105	-8.914	-6.194
DN45748_c0_g1_i3	ethylene-responsive transcription factor ERF027	-6.273	-8.585	-8.654
DN52615_c0_g1_i2	Fasciclin-like arabinogalactan 2	-2.648	-4.45	-3.728
DN57479_c2_g2_i5	gibberellin 2-β-dioxygenase 1	-3.804	-3.498	-4.008
DN45063_c0_g1_i1	glutamine synthetase, chloroplastic	-2.436	-3.216	-3.305
DN50563_c3_g1_i1	light-inducible CPRF-2	-2.388	-3.158	-3.113
DN55302_c1_g1_i1	NAC domain-containing 8	-5.379	-5.382	-5.088
DN53518_c2_g1_i2	NRT1 PTR FAMILY	-3.728	-4.567	-5.509
DN60796_c2_g1_i4	potassium channel AKT1	-3.261	-3.112	-3.271
DN50527_c1_g1_i1	vacuolar amino acid transporter 1	-3.593	-4.819	-5.168
DN57865_c2_g1_i1	wall-associated receptor kinase 1	-3.95	-3.625	-4.119
DN42518_c0_g1_i1	zinc finger AN1 domain-containing stress-associated 17	-3.782	-2.445	-2.894
DN57848_c0_g1_i1	zinc finger VAR3, chloroplastic	-2.881	-2.831	-3.214
DN63046_c3_g1_i2	α-expansin 5 isoform X1	-3.446	-3.493	-5.571

### Genes involved in abiotic stress responses

We analysed individual datasets for the up- and down-regulated DEGs per each stress treatment of DEGs (CULG, CURG, CDLG, CDRG, COSUG, and COSDG; see Fig 7B and 7E). Several genes that play an essential role in plant maintenance and development were transcriptionally affected due to osmotic stress conditions.

In CULG and CDLG sets (cores of Up- and down-regulated genes in leaf tissues respectively), we found several TFs families associated to stress responses like bZIP, ZF, HSF, NAC, MYB, PHR1, and AP2/ERF. All the TFs were regulated during the T1, T2, and T3 treatments (Table 2).

Only a few DEGs were found to be associated with secondary metabolism. In the current study, isoflavone 2-hydroxylase and chalcone synthase 1 markedly increased with osmotic stress imposition and reached the highest levels in T3 treatment. Several other genes were found to be importantly related to stress regulation (see Table 2).

Among the CURG and CDRG datasets (cores of Up- and down-regulated genes in root tissues respectively), we detected stress-related TFs representatives as NAC, HSF, MYB, bHLH, and AP2/ERF (Table 3). All of these TFs were significantly regulated during T1, T2, and T3 treatments. Others several stress related genes were regulated in root tissues after osmotic stress imposition like dehydrin DHN4 and dehydrin COR410, a cationic peroxidase SPC4, desiccation-related PCC13-62 protein, LEA 3 protein, BAX-Inhibitor protein, anthocyanidin reductase, among others.

**Table 3:** Stress-related DEGs detected in the CORE sets (CURG and CDRG) of regulated genes in roots during the osmotic stress treatments.

Gene code	Annotation	Gene expression In Root (Log2FC) T1 T2 T2		
DN61697_c2_g1_i1	9-cis-epoxycarotenoid dioxygenase 1, chloroplastic	6.599	4.27	4.341
DN58529_c2_g1_i1	anthocyanidin reductase	2.722	4.333	5.822
DN50090_c2_g1_i1	argonaute 2	5.926	4.622	4.395
DN57624_c2_g1_i3	cation H(+) antiporter 28	8.334	7.945	8.733
DN48722_c2_g2_i2	cationic peroxidase SPC4	5.787	4.769	4.426
DN50208_c0_g1_i1	chalcone synthase 1	2.844	5.212	6.316
DN58180_c0_g2_i2	dehydrin COR410	4.257	2.78	2.788
DN48173_c1_g1_i2	dehydrin DHN4	10.13	8.302	7.814
DN52301_c7_g1_i3	desiccation-related PCC13-62	7.931	8.664	8.082
DN53152_c3_g2_i1	ethylene-responsive transcription factor ERF053	4.471	3.863	4.24
DN62387_c7_g1_i1	ethylene-responsive transcription factor ERF113	4.554	4.374	4.237
DN57127_c4_g1_i15	ethylene-responsive transcription factor RAP2-3	9.189	8.256	9.249
DN55043_c4_g1_i6	heat stress transcription factor C-2a	3.226	3.145	3.015
DN59732_c7_g1_i2	laccase-15	5.138	7.365	8.993
DN52623_c0_g2_i2	late embryogenesis abundant, group 3	8.713	7.292	5.969
DN52494_c0_g1_i2	low-temperature-induced 65 kDa	6.484	5.962	6.451
DN49045_c1_g1_i1	NAC transcription factor 25	3.6	3.329	3.322
DN64098_c7_g1_i4	potassium transporter 5	5.401	4.932	6.154
DN63369_c4_g1_i2	probable phosphatase 2C 8	6.255	6.282	5.397
DN65258_c1_g1_i2	scarecrow 8	2.968	2.953	3.159
DN50725_c1_g1_i2	small heat shock, chloroplastic	3.976	4.317	4.309
DN54677_c4_g1_i2	SNF1-related kinase regulatory subunit α-1	5.796	5.208	5.278
DN57540_c2_g1_i1	transcription factor JUNGBRUNNEN 1	2.782	3.009	3.291
DN55589_c3_g1_i1	transcription factor MYB108	4.875	4.569	4.352
DN53533_c4_g1_i1	transmembrane BAX inhibitor motif-containing 4	4.598	4.019	4.586
DN59092_c9_g2_i1	universal stress A	4.49	4.191	5.055
DN51942_c0_g1_i2	zinc finger ZAT8	4.036	3.114	3.001
DN45438_c0_g1_i3	abscisic stress-ripening 3	-7.321	-4.563	-5.747
DN47034_c3_g1_i5	aquaporin TIP2-3	-3.531	-4.183	-4.39
DN61165_c0_g1_i2	auxin response factor 1	-2.468	-3.212	-3.043
DN37737_c0_g1_i2	bidirectional sugar transporter SWEET3a	-8.706	-6.264	-8.749
DN43633_c0_g1_i1	bidirectional sugar transporter SWEET11	-6.168	-8.759	-8.777
DN45479_c0_g2_i2	casparian strip membrane 2	-8.359	-8.419	-8.439
DN60501_c0_g1_i1	cellulose synthase E6	-2.371	-2.329	-2.557
DN50424_c7_g1_i1	dehydration-responsive element-binding 1G	-3.76	-3.675	-4.616
DN64458_c0_g1_i1	DNA (cytosine-5)-methyltransferase 1	-4.899	-5.213	-5.03
DN45748_c0_g1_i3	ethylene-responsive transcription factor ERF027	-7.879	-7.928	-7.946
DN64662_c1_g1_i1	NETWORKED 1A	-2.791	-2.287	-2.46
DN49943_c2_g1_i1	ROOT HAIR DEFECTIVE 3	-3.7	-2.448	-2.415
DN42499_c2_g1_i1	senescence-associated DIN1	-4.047	-4.429	-3.046
DN52278_c3_g1_i10	sugar transporter ERD6 16	-2.287	-4.196	-3.065
DN57037_c0_g1_i11	topless-related 1	-2.766	-3.523	-3.707
DN47439_c0_g1_i1	transcription factor bHLH94	-5.08	-6.95	-6.956
DN57431_c0_g1_i1	transcription factor MYB59	-2.922	-3.118	-4.306
DN57534_c11_g1_i4	vacuolar amino acid transporter 1	-3.022	-2.458	-3.124
DN45850_c1_g2_i1	wall-associated receptor kinase 2	-4.345	-4.982	-5.607
DN48034_c1_g1_i1	WALLS ARE THIN 1	-4.155	-4.118	-4.485

Some genes down-regulated by effect of the stress imposition were: bidirectional sugar transporters, casparian strip membrane 2, wall-associated receptor kinase 2, ROOT HAIR DEFECTIVE 3, cellulose synthase E6, DNA (cytosine-5)-methyltransferase 1, among others (see Table 3).

Selected genes (NAC-25 TF, bidirectional sugar transporter SWEET6a, PIP2-1, and Abscisic Acid 8-hidroxilase 3) were selected for RT-qPCR analysis to confirm the reproducibility of gene profiles detected by bioinformatic analysis (S2 Fig). NAC-25 TF, bidirectional sugar transporter SWEET6a, PIP2-1, and Abscisic Acid 8-hidroxilase 3 (this gene was used for qRT-PCR analysis in leaf and root tissues). Although the exact expression pattern of selected genes varied among the RT-qPCR and RNA-Seq analysis, their expression was quite similar in both cases.

### GO functional analysis for DEGs

**Fig 8:**
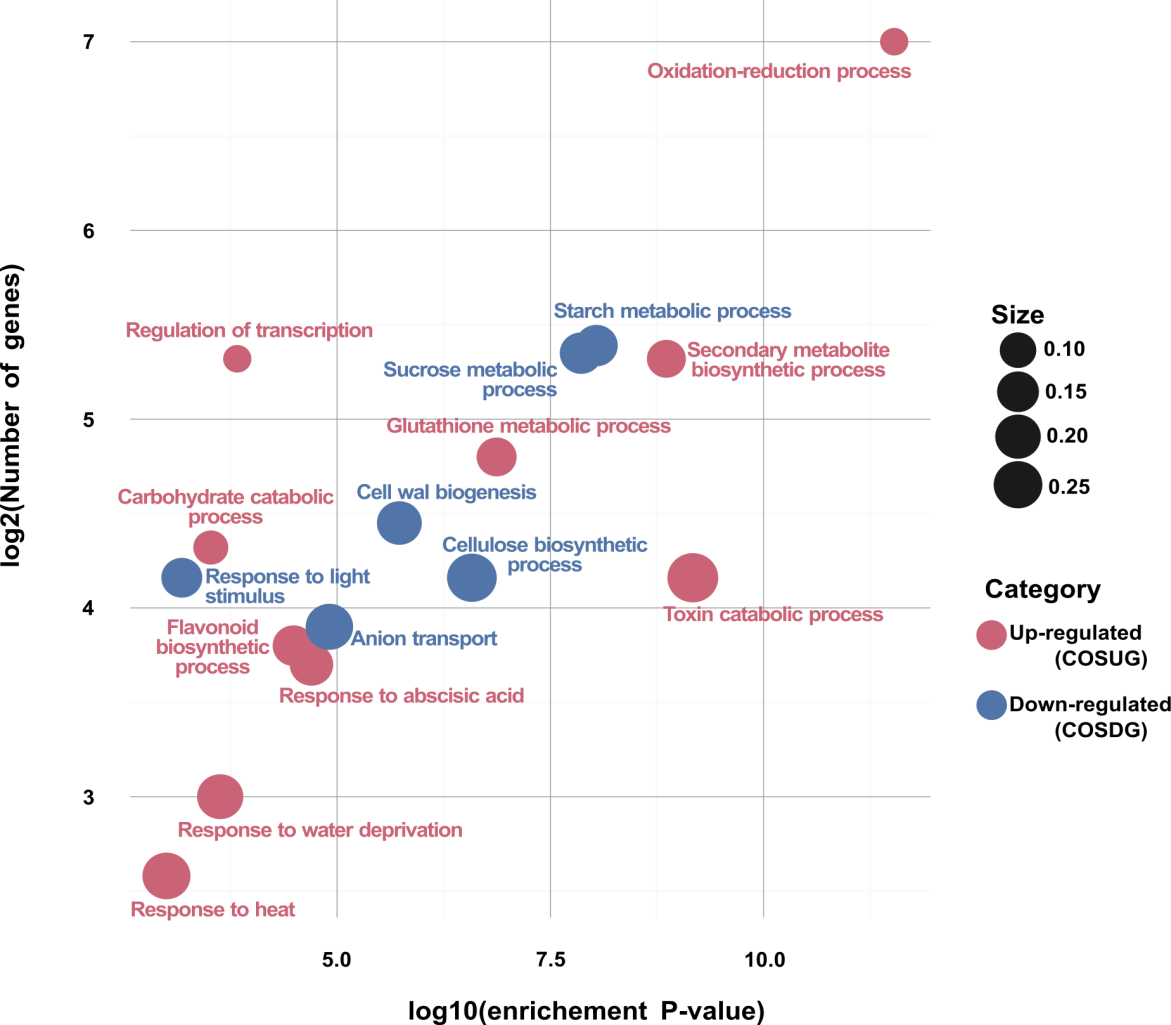
Functional enrichment analysis for the CORE set of DEGs regulated in the osmotic stress treatments for sugarcane var. Mex 69–290. Significant groups (FDR<0.05) for COSUG and COSDG groups are plotted according to their enriched adjusted p-value (x axis). The size is proportional to the number of enriched genes among the total number of their GO term associated unigenes. COSUG = CORE of Osmotic Stress Up-regulated Genes for both leaf and root tissues, COSDG = CORE of Osmotic Stress Down-regulated Genes for both leaf and root tissues.

All the DEGs were annotated against the GO database (http://www.geneontology.org/) by Blast2GO software [21], to reveal significantly enriched GO terms in DEGs, besides, we also divided these terms into up-regulated and down-regulated groups. The GO enrichment analysis was conducted for each up and a down-regulated set of genes for leaf and root tissues using the Fisher’s Exact Test. The results were subsequently adjusted using a FDR< 0.05 correction. We further analysed the functional enrichment for the COSUG and COSDG groups (Fig 8).

As shown in Fig 8, response to heat, water deprivation, abscisic acid, secondary metabolite biosynthetic process (as flavonoids), regulation of transcription, glutathione metabolic processes, among others, were the main enriched GO terms for up-regulated DEGs. On the other hand, response to light stimulus, anion transport, sucrose and starch metabolic processes, cellulose biosynthetic processes, among others, were the primary enriched GO terms for down-regulated core of DEGs.

## Discussion

### Physiological responses of sugarcane under osmotic stress treatments

Tissue culture is a great tool for the assessment of many hypotheses because a homogenous population of plants can be easily controlled, as well as the conditions of the assay [22, 23]. In this context, PEG has been used for several groups to mimic drought conditions *in vitro* and *ex vitro* [22, 24–28]. PEG is a non-penetrating, inert, and non-phytotoxic agent that reduces the osmotic potential of culture medium inducing an osmotic stress in plant cells. Nevertheless, an outdoor evaluation of the in vitro tested condition is needed for a better understanding of the real and more complex processes that are triggered by drought conditions [29].

In this study, we describe the response of 90 days-old sugarcane plantlets after severe osmotic stress. The decrease in RWC is dependent to the time of stress imposition and reached its lowest value in T3 treatments with 31.17% less than T0. Comparatively, [30] reported a decrease of 25% in the RWC during mild stress (3 DWW; days by withholding water) and 33% during severe stress (9 DWW).

Sugarcane plantlets were significantly affected in CO_2_ assimilation and transpiration rate after 48 h of osmotic stress. This observation can be explained by taking into account that drought conditions prevent CO_2_ from entering the leaves, influence absorption of CO_2_ by carboxylation centre result in a decrease of net photosynthetic rate [31].

Over production of proline results in augmentation level tolerance to osmotic stress in plants. Here, proline content increased in both leaves and roots along the stress. Similar physiological parameters were reported in [30] for sugarcane variety GT21, showing significant alterations in response to drought stress. Our physiological data suggest a significant change in the regulation of molecular mechanisms that trigger the physiological responses caused by sensing the osmotic stress imposed by PEG 8000.

### The sugarcane transcriptome

The osmotic stress is a principal component of many abiotic stresses, which interferes with the capability of plants to sense cell wall damage due to reduced turgor originated in an inappropriate activation of defence mechanisms [32]. Recently, important efforts have been made towards the identification of stress-regulated genes aiming to improve the resilience capability against drought in economically important crops [33–35].

Rocha *et al*. reported 93 differentially expressed genes in sugarcane plants exposed to drought using macro-array technology which contained 1,545 probes [10]; among the DEG were hsp70, expansines, tyrosine phosphatases, catalases, Fructose-bisphosphate aldolase, trehalose metabolism-related proteins, most of which were found in our analysis. Similar to [10], Rodrigues and collaborators found 165 DEG associated to water deficit by using macroarray membranes containing 3,575 probes [11]; among these DEG were found DNAj proteins, PP2C, AP2 transcription factors, calmodulins, peroxidases, hsp70, Fructose-bisphosphate aldolase, among many others, similar to our results. Li and collaborators reported that 1,501 genes were differentially regulated (9.6% of the tested genes) in leaves under water-deficit by using a microarray technique [30], many of them involved in metabolic pathways such as biosynthesis of secondary metabolites, ribosomes, carbon metabolism, among others.

In the present study, a total of 11,796 and 8,611 genes were identified as DEG in leaf and root tissues, respectively. The PCA and the hierarchical clustering showed a tissue-specific expression pattern of the DEGs. The PCA and HCA revealed a significant change in gene expression after the first 24 h of stress imposition for both tissues, and these changes were specific according to the tissues. These results are visually evident on our heatmap (Fig 5A). The non-stressed tissue samples had very similar expression patterns in comparison to the stressed samples. The clusters E, F, and G (with 452, 161, and 31 genes; Fig 5B) showed a rapid change in the expression patterns starting at T1 treatment, and with little changes for T2 and T3 stressed samples.

### DEGs and pathways regulated by osmotic stress conditions

The GO enrichment analysis shows that several of the up-regulated sequences were grouped into many stress-related pathways as response to abscisic acid, water deprivation, heat, secondary metabolite biosynthesis, oxidation-reduction process, cellular chemical homeostasis, regulation of transcription, among others. On the other hand, several down-regulated sequences were grouped into photosynthesis related pathways as photosynthesis-dark reaction, chlorophyll biosynthetic process, and photosynthesis-light harvesting in photosystem I. Furthermore, some important carbohydrate-related pathways were enriched such as starch metabolic process, sucrose metabolic process, cellulose biosynthetic process, cell wall biogenesis, regulation of transcription, among others (Fig 8).

These pathways are of great interest since the most economically important characteristic of sugarcane is its biomass production and accumulation of sucrose [36]. The results show negative effects of osmotic stress on biosynthesis of sucrose [37]. Being a high water-demanding crop, the major limiting factors in sugarcane cultivation are abiotic stresses such as drought, salinity, and extreme temperatures [38].

We detected expresion of strees related genes like Dehydrins and LEA proteins [39], antioxidant proteins as peroxidases and catalases [40], detoxification proteins as glutathione reductase [41], transporters [42], heat shock proteins [43], secondary metabolite biosynthesis [44], and TFs [45].

Comparatively, data mining analysis for sugarcane done by using the Sugarcane Expressed Sequence Tags (SUCEST) database, revealed an abundant expression of genes encoding chaperones, co-chaperones and other proteins linked to protection against stress [46].

**Fig 9:**
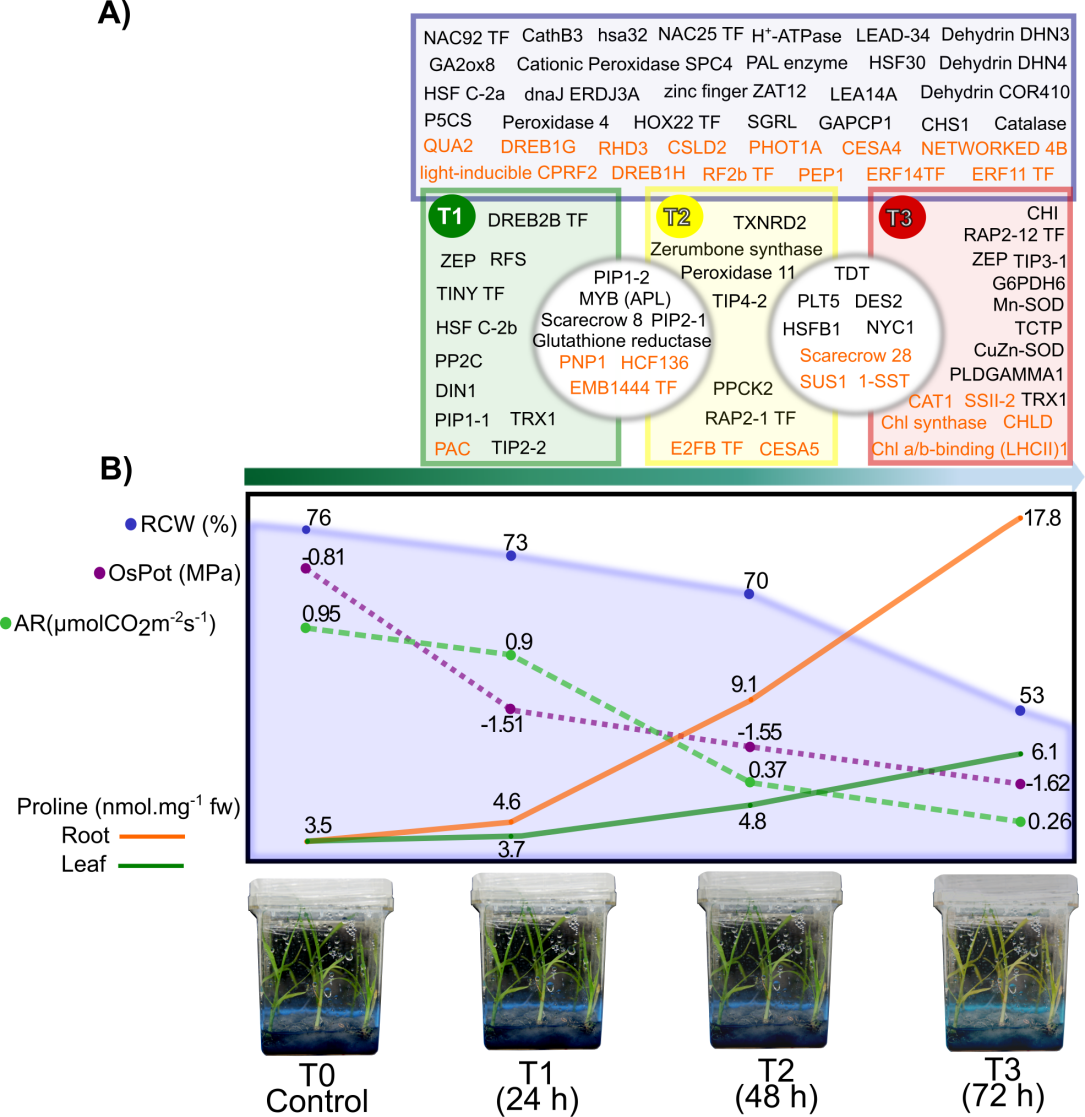
Principal findings of the analysis of sugarcane transcriptome under osmotic stress. A) Genes regulated under osmotic stress treatments. Inside blue square are the DEGs regulated in the three treatments. The green square represents the DEG regulated only in the T1 treatment, within the yellow square are the DEGs expressed only in T2 treatment, and within the red square are the DEGs regulated only in T3 treatment. Inside gray circles are DEGs shared among treatments. Letters in black color represent the up-regulated DEGs and letters in orange color represent the down-regulated DEGs per treatment. B) Schematic depiction of the physiological results obtained in sugarcane var. Mex 69–290, imposed to an osmotic stress treatment by PEG 8000 for 24, 48, and 72 h. The green arrow under the depiction indicates the directions of the time-stress treatments. RCW = relative content water, OsPot = osmotic potential, AR = assimilation rate. Complete name of the genes in A) are listed in the S4 File.

A well represented group of coding genes found in our transcriptome is the group of LEA proteins, commonly modulated by ABA [47]. Some LEA proteins have been associated with diverse specific functions as anti-aggregation activity, membrane stabilization, ion scavenging, metal binding and water sequestration, among others [48]. Along the kinetic, some members of this group of proteins were detected, for example LEA14A, Dehydrin DHN3, Dehydrin DHN4 and COR410 (see Fig 9).

Transcription factors are another interesting group of genes involved in osmotic stress that were enriched in our analysis. Among the up-regulated TFs in sugarcane, we detected three principal representative groups belonging to the HSF, NAC, and AP2/ERF families, previously reported as stress-responsive TFs [49]. The transcript level of P5CS, an important gene of the proline biosynthesis pathway, was significantly altered upon exposure to PEG stress, in contrast with the reported by [50]. However, induction of transcript expression of OsP5CS was reported in rice in response to high salt, dehydration, ABA and cold treatments [51].

Osmotic stress conditions also enhance the production of reactive oxygen species (ROS) that propel the cellular damage inducing oxidative stress. All pathways that activate mechanisms leading to ROS scavenging have been shown to play an important role in protecting plants against different abiotic stresses [52]. Several genes coding detoxifying enzymes related to the response against oxidative stress are triggered by osmotic stress to mitigate the potential damage induced by ROS and other reactive. Other important groups of proteins involved in the redox metabolism are the thioredoxins, thiol-disulfide oxidoreductase enzymes participating in the cellular signalling pathways mediated by redox mechanism.

All these groups of proteins were subjected to regulation at transcriptional level in the experimental conditions of the present study, suggesting an induction of detoxifying systems in response to a parallel oxidative stress.

Additionally to the characterized and identified transcripts, a total of 20.2% of the 20,407 DEGs showed an unknown function (no match + uncharacterized protein). Li *et al*., (2016) determined by using microarray analysis that ~38.7% of DEG were assigned as proteins with unknown functions [30]. In accordance, this finding remarks the importance of the study of proteins with assigned unknown function, as a benchmark for the discovery of new genetic sources for the understanding of molecular signalling during drought phenomenon.

## Conclusions

The lack of genome and transcriptome data has restricted molecular studies in *S*. *officinarum*. Here, we provide a benchmark set of transcript profiles responding to osmotic stress. The knowledge gained from these studies play an important role on the grasp of the molecular processes involved in the osmotic stress response in sugarcane. The next challenge is to test those drought responsive genes in monocot plant models as rice as well as sugarcane itself, to evaluate the functionality of the selected genes. Finally, the development of stress tolerant sugarcane cultivars will be substantially beneficial for the production of this crop in drought predicted scenarios.

## Methods

### Plant material and experimental design

*S*. *officinarum*, var. Mex 69–290 was regenerated via somatic embryogenesis described by [53].

Osmotic stress was induced in *in vitro*-plantlets (90-days) which were transferred to magenta dishes containing liquid MS media and enriched with 40% of PEG 8000 (Sigma-Aldrich®) This PEG 8000 concentration corresponds to an osmotic potential of -4.5 MPa, measured with a digital hygrometer (Bapro®).

### Tissue collection and total RNA extraction

RNA extraction from *S*. *officinarum* aerial tissues (stem and leaves) and roots was carried out using TRIzol reagent (Invitrogen®) according to the manufacturer's instructions. The integrity and quantity of the purified RNA were assessed both on 1% agarose gel and 2100 Bioanalyzer (Agilent Technologies®). Equal quantities of high-quality RNA from each sample were pooled for the library preparation.

### Library construction, RNA-seq raw data cleaning, and assembly

Libraries were constructed and sequenced at the Genomic Services Laboratory, (LANGEBIO-CINVESTAV, Mexico). Libraries were prepared with TruSeq RNA Sample Prep Kit v2 (Illumina), and library quantification was performed with a 2100 Bioanalyzer (Agilent Technologies®). For high-throughput sequencing, paired-end sequence samples were multiplexed with 16 libraries in one line, yielding ~410 million clean reads and with ~22 million reads per library for *S*. *officinarum* transcriptome. All libraries were sequenced in a 2x100 bp configuration on the HiSeq2500 Illumina platform. Raw reads were checked for quality with FASTQC (http://www.bioinformatics.babraham.ac.uk/projects/fastqc/) [54], processed by clipping adapters, cleaned and filtered for quality scores greater than 30 and read length greater than 80 nucleotides using the Trimmomatic v0.36 toolkit [55].

### Generating a reference transcriptome of sugarcane

The transcriptome reference was obtained from a RNA mix from leaves, stems and roots of plantlets of sugarcane var. Mex 69–290. RNA-seq was carried out on the complementary DNA libraries derived from non-stressed plantlets and groups of plantlets subjected to 24, 48, and 72 h of osmotic stress. Four libraries for leaves+stems (L0, L1, L2, L3; L = leaf) and four libraries for roots (R0, R1, R2, and R3; R = root) were sequenced with their respective biological replicates. A total, 412,440,102 raw reads were generated, and 410,382,788 clean reads with a Q>30 were retained. *De novo* assembly of all QC cleared reads was conducted with Trinity software v2.2.0 [56] and following the protocol reported in [57]. The number of unigenes, N50 statistics, and the average coverage were used to evaluate the sequence assembly quality (see Table 1).

All unigenes were annotated by assigning their associated generic Gene Ontology (GO) terms and Enzymes codes (EC) using Blast2GO software [21]. The annotation was based on the homology to proteins from other plant species determined by BLASTX. Plant ref-seq homology searches were complemented with information from INTERPROSCAN [58], the Kyoto Encyclopedia of Genes and Genomes (KEGG; [19] biochemical pathways, and expanded using ANNEX [59]. BLAST searches of the unigenes were conducted against Swiss-Prot reviewed database (www.uniprot.org) containing 551,705 manual annotated proteins and TrEMBL database (www.uniprot.org) with 65,378,749 un-reviewed proteins. TransDecoder v3.0.0 software was used to identify candidate coding regions within unigene sequences generated by our *de novo* RNA-Seq transcript assembly, yielding a total of 94,416 open reading frames (ORFs) of at least 100 amino acids long. Detected ORFs were used for homology searches against Pfam-A v9.0 database [60] containing 5,724 families and 705,698 proteins, and another search was conducted against the Conserved Domain Database (CDD) [61] by the Batch CD-Search tool [62]. The original KEGG map used for depict the starch and sucrose metabolism genes in Fig 3 is found in S5 File.

### Differential expression analysis and GO annotation

Expression abundances were determined by mapping the pair-end read libraries (each replicate for each tissue) independently against our *S*. *officinarum* de novo transcriptome, using RSEM software v1.2.27 (RNA-Seq by Expectation-Maximization [20], implemented with bowtie aligner [63]). Expression was normalized to a transcripts per million transcripts (TPM), representing gene expression level. Nevertheless, the implemented script calculates both TPM and FPKM (Fragments per kilobase of exon per million fragments mapped) values. For a detailed explanation of all the steps and scripts implemented in this work (assembly, filtering, comparisons, gene clustering methods, and differential expression analysis) please see [57]. The implemented pipeline is available at the github repository (https://github.com/trinityrnaseq/trinityrnaseq.wiki.git).

DEGs between treatments and tissues was determined by EdgeR [64] in R [65] following the pipeline described in [57]. For each gene, a significant threshold of 0.001 was applied after the p-value was adjusted with false discovery rate (FDR) via Bonferroni-Holms correction [66]. For the prediction analysis of gene homology, sugarcane DEGs were aligned to the Plant ref-seq proteins from NCBI with a 1e^-3^ E-value threshold by using BLASTX to annotate gene functions. Functional annotations were also created by Blast2GO software [21] with the GO and EC terms, and KEGG EC biochemical pathways.

### Data and statistical analysis

Data analysis and graphics were performed with the R statistical package [65] unless stated otherwise. All the graphs were done by ggplot2 [67] and Vennerable R packages (https://github.com/js229/Vennerable). To provide statistical support for physiological and biochemical analyses, we use the Tuckey’s HSD (Honest Significance Difference; p<0.05) test for each treatment by using STATGRAPHICS Centurion XVI.I software and SigmaPlot 11.0 software. Figures editing were done by Inkscape v0.91 software (www.inkscape.org).

### Gene validation by RT-qPCR

Five selected candidate genes were evaluated by quantitative real-time PCR. The designed primers used on the analysis are on S1 Table. The relative quantification of each transcript was calculated by the 2^-ΔΔC^_T_ method using ACT as internal control [68].

### Determination of physiological parameters

Photosynthesis rate was determined with a portable Li-6400 photosynthetic system (LI-COR Biosciences®), using three samples per treatment. Plant water status was determined by measuring the osmotic potential on fully expanded leaves with a thermocouple psychrometer (HR-33T Dew point microvoltmeter, sample chambers type C-52; USA). RWC was computed using the formula in [69] RWC = [(FW—DW)/(TW—DW)] x 100, using the flag leaf of four plantlets. Proline content determination was performed for root and leaves according to [70].

### Availability of data and materials

The RNA-seq datasets supporting the conclusions of this article were deposited in the National Center of Biotechnology Information (NCBI) under the Sequence Read Archive (SRA) repository, https://www.ncbi.nlm.nih.gov//bioproject/PRJNA371469, under accession numbers SRR5239234-SRR5239249.

## Supporting information

S1 Table List of sugarcane genes and primers used for the RT-qPCR validation. (PDF)

S1 Fig Pearson's correlation analysis among replicates of each biological sample. (PDF)

S2 Fig RT-qPCR analysis of five significatively regulated genes randomly selected into the osmotic stress treatments. (PDF)

S1 File Gene Ontology terms of all the mapped unigenes. (XLSX)

S2 File Unigenes found against the Kyoto Encyclopedia of Genes and Genomes (KEGG) biochemical pathways. (XLSX)

S3 File Copyright permission of Fig 3. (PDF)

S4 File Abbreviations and acronyms. (PDF)

S5 File Original KEGG map used for depict the starch and sucrose metabolism genes in Fig 3. (PNG)

## References

[1] Lakshmanan P, Geijskes RJ, Aitken KS, Grof CPL, Bonnett GD, Smith GR. Sugarcane biotechnology: The challenges and opportunities. In Vitro Cellular & Developmental Biology—Plant. 2005; 41: 345–363.

[2] Waclawovsky AJ, Sato PM, Lembke CG, Moore PH, Souza GM. Sugarcane for bioenergy production: an assessment of yield and regulation of sucrose content. Plant Biotechnology Journal. 2010; 8: 263–276. doi: 10.1111/j.1467-7652.2009.00491.x 20388126 10.1111/j.1467-7652.2009.00491.x

[3] Kim JY, Gallo M, Altpeter F. Analysis of transgene integration and expression following biolistic transfer of different quantities of minimal expression cassette into sugarcane (*Saccharum spp*. hybrids). Plant Cell, Tissue and Organ Culture. 2012; 108: 297–302.

[4] Tammisola J. Towards much more efficient biofuel crops—can sugarcane pave the way? Genetically Modified Crops. 2010; 1: 181–198. 10.4161/gmcr.1.4.13173 21844673

[5] Cha-um S, Kirdmanee C. Effect of osmotic stress on proline accumulation, photosynthetic abilities and growth of sugarcane plantlets (*Saccharum officinarum* L.). Pakistan journal of botany. 2008; 40: 2541–2552.

[6] Zhao D, Li Y. Climate change and sugarcane production: potential impact and mitigation strategies. International Journal of Agronomy. 2015; http://dx.doi.org/10.1155/2015/547386

[7] Ortega-Gaucin D, Lopez-Perez M, Arreguin-Cortez FI. Drought risk management in Mexico: Progress and challenges. International Journal of Safety and Security Engineering. 2016; 6: 161–170.

[8] Moyer M. How much is left? A graphical accounting of the limits to what one planet can provide. Scientific American. 2010; 303: 74–81. 10.1038/scientificamerican0910-74 20812483

[9] Esperón-Rodríguez M, Bonifacio-Bautista M, Barradas VL. Socio-economic vulnerability to climate change in the central mountainous region of eastern Mexico. Ambio. 2016; 45: 146–160. doi: 10.1007/s13280-015-0690-4 26224563 10.1007/s13280-015-0690-4 PMC4752563

[10] Rocha FR, Papini-Terzi FS, Nishiyama MY Jr, Vêncio RZN, Vicentini R, Duarte RDC, et al Signal transduction-related responses to phytohormones and environmental challenges in sugarcane. BMC Genomics. 2007; 8:71 doi: 10.1186/1471-2164-8-71 17355627 10.1186/1471-2164-8-71 PMC1852312

[11] Rodrigues FA, Laia ML, Zingaretti SM. Analysis of gene expression profiles under water stress in tolerant and sensitive sugarcane plants. Plant Science. 2009; 176: 286–302.

[12] Rodrigues FA, Da Graça JP, De Laia ML, Nhani A Jr, Galbiati JA, Ferro MIT, et al Sugarcane genes differentially expressed during water deficit. Biologia Plantarum. 2011; 55: 43–53.

[13] Lembke CG, Nishiyama MY Jr, Sato PM, De Andrade RF, Souza GM. Identification of sense and antisense transcripts regulated by drought in sugarcane. Plant Molecular Biology. 2012; 79: 461–477. doi: 10.1007/s11103-012-9922-1 22610347 10.1007/s11103-012-9922-1 PMC3369129

[14] Begcy K, Mariano ED, Gentile A, Lembke CG, Zingaretti SM, Souza GM, et al A novel stress-induced sugarcane gene confers tolerance to drought, salt and oxidative stress in transgenic tobacco plants. PLoS ONE. 2012; https://doi.org/10.1371/journal.pone.0044697 10.1371/journal.pone.0044697 PMC3439409 22984543

[15] Metzker ML. Applications of next-generation sequencing technologies: The next generation. Nature Reviews Genetics. 2010; 11: 31–46. doi: 10.1038/nrg2626 19997069 10.1038/nrg2626

[16] Imadi SR, Kazi AG, Ahanger MA, Gucel S, Ahmad P. Plant transcriptomics and responses to environmental stress: an overview. Journal of Genetics. 2015; 94: 525–537. 26440096 10.1007/s12041-015-0545-6

[17] Cardoso-Silva CB, Costa EA, Mancini MC, Balsalobre TWA, Costa-Canesin LE, Pinto LR, et al De novo assembly and transcriptome analysis of contrasting sugarcane varieties. PLoS ONE. 2014; https://doi.org/10.1371/journal.pone.0088462 10.1371/journal.pone.0088462 PMC3921171 24523899

[18] Mattiello L, Riaño-Pachón DM, Mattos Martins MC, Prado da Cruz L, Bassi D, Ribeiro Marchiori PE, et al Physiological and transcriptional analyses of developmental stages along sugarcane leaf. BMC Plant Biology. 2015; 15:300 doi: 10.1186/s12870-015-0694-z 26714767 10.1186/s12870-015-0694-z PMC4696237

[19] Kanehisa M, Furumichi M, Tanabe M, Sato Y, Morishima K. KEGG: new perspectives on genomes, pathways, diseases and drugs. Nucleic Acids Research. 2017; 45: D353–D361. doi: 10.1093/nar/gkw1092 27899662 10.1093/nar/gkw1092 PMC5210567

[20] Li B, Dewey CN. RSEM: accurate transcript quantification from RNA-Seq data with or without a reference genome. BMC Bioinformatics. 2011; 12:323 doi: 10.1186/1471-2105-12-323 21816040 10.1186/1471-2105-12-323 PMC3163565

[21] Conesa A, Gotz S, Garcia-Gomez JM, Terol J, Talon M, Robles M. Blast2GO: a universal tool for annotation, visualization and analysis in functional genomics research. Bioinformatics. 2005; 21: 3674–3676. doi: 10.1093/bioinformatics/bti610 16081474 10.1093/bioinformatics/bti610

[22] Munir N, Aftab F. The role of polyethylene glycol (PEG) pretreatment in improving sugarcane’s salt (NaCl) tolerance. Turkish Journal of Botany. 2009; 33, 407–415.

[23] Errabii T, Gandonou CB, Essalmani H, Abrini J, Idaomar M, Skali-senhaji N. Growth, proline and ion accumulation in sugarcane callus cultures under drought-induced osmotic stress and its subsequent relief. African Journal of Biotechnology. 2006; 5: 1488–1493.

[24] Kaufmann MR, Eckard AN. Evaluation of water stress control with polyethylene glycols by analysis of guttation. Plant physiology. 1971; 47: 453–456. 16657642 10.1104/pp.47.4.453 PMC396708

[25] Zhou G, Yang LT, Li YR, Zou CL, Huang LP, Qiu LH, et al Proteomic analysis of osmotic stress-responsive proteins in sugarcane leaves. Plant Molecular Biology Reports. 2012; 30: 349–359.

[26] Zhang HM, Zhang LS, Lv H, Yu ZY, Zhang DP, Zhu WN. Identification of changes in Triticum aestivum L. leaf proteome in response to drought stress by 2D-PAGE and MALDI-TOF/TOF mass spectrometry. Acta Physiologiae Plantarum. 2014; 36: 1385–1398.

[27] Zhang M, Lv DW, Ge P, Bian YW, Chen GX, Zhu GR, et al Phosphoproteome analysis reveals new drought response and defense mechanisms of seedling leaves in bread wheat (Triticum aestivum L.). Journal of Proteomics. 2014; 109: 290–308. doi: 10.1016/j.jprot.2014.07.010 25065648 10.1016/j.jprot.2014.07.010

[28] Rabey HAE, Al-Malki AL, Abulnaja KO. Proteome Analysis of Date Palm (Phoenix dactylifera L.) under Severe Drought and Salt Stress. International Journal of Genomics. 2016; 7840759: 1–8. 10.1155/2016/7840759 PMC5093262 27840818

[29] Zhao F, Zhang D, Zhao Y, Wang W, Yang H, Tai F, et al The difference of physiological and proteomic changes in maize leaves adaptation to drought, heat, and combined both stresses. Frontiers in Plant Science. 2016; 7: 1471 doi: 10.3389/fpls.2016.01471 27833614 10.3389/fpls.2016.01471 PMC5080359

[30] Li C, Nong Q, Solanki MK, Liang Q, Xie J, Liu X, et al Differential expression profiles and pathways of genes in sugarcane leaf at elongation stage in response to drought stress. Scientific Reports. 2016; 6: 1–11. 27170459 10.1038/srep25698 PMC4864372

[31] Zhang SR. A discussion on chlorophyll fluorescence kinetics parameters and their significance. Chinese Bulletin of Botany. 1999; 16: 444–448.

[32] Kissoudis C, van de Wiel C, Visser RGF, van der Linden G. Enhancing crop resilience to combined abiotic and biotic stress through the dissection of physiological and molecular crosstalk. Frontiers in Plant Science. 2014; 5:207 doi: 10.3389/fpls.2014.00207 24904607 10.3389/fpls.2014.00207 PMC4032886

[33] Jogaiah S, Govind SR, Tran LS. Systems biology-based approaches toward understanding drought tolerance in food crops. Critical Reviews in Biotechnology. 2013; 33: 23–39. doi: 10.3109/07388551.2012.659174 22364373 10.3109/07388551.2012.659174

[34] Urban MO, Vašek J, Klíma M, Krtková J, Kosová K, Prášil IT, et al Proteomic and physiological approach reveals drought-induced changes in rapeseeds: Water-saver and water-spender strategy. Journal of Proteomics. 2017; 152: 188–205. doi: 10.1016/j.jprot.2016.11.004 27838467 10.1016/j.jprot.2016.11.004

[35] Wang X, Cai X, Xu C, Wang Q, Dai S. Drought-responsive mechanisms in plant leaves revealed by proteomics. International Journal of Molecular Sciences. 2016; 17(10): 1706 doi: 10.3390/ijms17101706 27763546 10.3390/ijms17101706 PMC5085738

[36] Lam E, Shine J, Da Silva J, Lawton M, Bonos S, Calvino M, et al Improving sugarcane for biofuel: engineering for an even better feedstock. GCB Bioenergy. 2009; 1: 251–255.

[37] Iskandar HM, Casu RE, Fletcher AT, Schmidt S, Xu J, Maclean DJ, et al Identification of drought-response genes and a study of their expression during sucrose accumulation and water deficit in sugarcane culms. BMC Plant Biology. 2011; 11: doi: 10.1186/1471-2229-11-12 21226964 10.1186/1471-2229-11-12 PMC3030532

[38] Sruthy MA, Narayan JA, Syamaladevi DP, Appunu C, Chakravarthi M, Ravichandran M, et al Introduction of Pea DNA Helicase 45 into Sugarcane (Saccharum spp. Hybrid) Enhances Cell Membrane Thermostability and Upregulation of Stress-Responsive Genes Leads to Abiotic Stress Tolerance. Molecular Biotechnology. 2015; 57: 475–488. doi: 10.1007/s12033-015-9841-x 25875731 10.1007/s12033-015-9841-x

[39] Hanin M, Brini F, Ebel C, Toda Y, Takeda S, Masmoudi K. Plant dehydrins and stress tolerance. Versatile proteins for complex mechanisms. Plant Signaling & Behavior. 2011; 6: 1503–1509. 21897131 10.4161/psb.6.10.17088 PMC3256378

[40] Miller AF. Superoxide dismutases: Ancient enzymes and new insights. FEBS Letters. 2012; 586: 585–595. doi: 10.1016/j.febslet.2011.10.048 22079668 10.1016/j.febslet.2011.10.048 PMC5443681

[41] Kabir AH, Khatun MA, Hossain MM, Haider SA, Alam MF, Paul NK. Regulation of phytosiderophore release and antioxidant defense in roots driven by shoot-based auxin signaling confers tolerance to excess iron in wheat. Frontiers in Plant Science. 2016; 7: 1–15. 27891139 10.3389/fpls.2016.01684 PMC5103167

[42] Reinders A, Panshyshyn JA, Ward JM. Analysis of Transport Activity of Arabidopsis Sugar Alcohol Permease Homolog AtPLT5. The Journal of Biological Chemistry. 2005; 280: 1594–1602. doi: 10.1074/jbc.M410831200 15525644 10.1074/jbc.M410831200

[43] Borges JC, Peroto MC, Ramos CHI. Molecular chaperone genes in the sugarcane expressed sequence database (SUCEST). Genetics and Molecular Biology. 2001; 24: 85–92.

[44] Schwarz N, Armbruster U, Iven T, Brückle L, Melzer M, Feussner I, et al Tissue-specific accumulation and regulation of zeaxanthin epoxidase in Arabidopsis reflect the multiple functions of the enzyme in plastids. Plant Cell Physiology. 2015; 56: 346–357. doi: 10.1093/pcp/pcu167 25416291 10.1093/pcp/pcu167

[45] Li M, Liang Z, Zeng Y, Jing Y, Wu K, Liang J, et al De novo analysis of transcriptome reveals genes associated with leaf abscission in sugarcane (*Saccharum officinarum* L.). BMC Genomics. 2016; 17:195 doi: 10.1186/s12864-016-2552-2 26946183 10.1186/s12864-016-2552-2 PMC4779555

[46] De Andrade JCF, Terto J, Silva JV, Almeida C. Expression profiles of sugarcane under drought conditions: Variation in gene regulation. Genetics and Molecular Biology. 2015; 38: 465–469. doi: 10.1590/S1415-475738420140288 26537606 10.1590/S1415-475738420140288 PMC4763319

[47] Dalal M, Tayal D, Chinnusamy V, Bansal KC. Abiotic stress and ABA-inducible group 4 LEA from *Brassica napus* plays a key role in salt and drought tolerance. Journal of Biotechnology. 2009; 139: 137–145. doi: 10.1016/j.jbiotec.2008.09.014 19014980 10.1016/j.jbiotec.2008.09.014

[48] Popova AV, Rausch S, Hundertmark M, Gibon Y, Hincha DK. The intrinsically disordered protein LEA7 from Arabidopsis thaliana protects the isolated enzyme lactate dehydrogenase and enzymes in a soluble leaf proteome during freezing and drying. Biochimica et Biophysica Acta. 2015; 1854: 1517–1525. doi: 10.1016/j.bbapap.2015.05.002 25988244 10.1016/j.bbapap.2015.05.002

[49] Lata C, Muthamilarasan M, Prasad M. Drought stress responses and signal transduction in plants In: Pandey KG, editor. Elucidation of Abiotic Stress Signaling in Plants: Functional Genomics Perspectives, vol 2 New York: Springer; 2015 pp. 195–225.

[50] Patade VY, Bhargava S, Suprasanna P. Transcript expression profiling of stress responsive genes in response to short-term salt or PEG stress in sugarcane leaves. Molecular Biology Reports. 2012; 39: 3311–3318. doi: 10.1007/s11033-011-1100-z 21706351 10.1007/s11033-011-1100-z

[51] Igarashi Y, Yoshiba Y, Sanada Y, Yamaguchi-Shinozaki K, Wada K, Shinozaki K. Characterization of the gene for D1-pyrroline-5-carboxylate synthetase and correlation between the expression of the gene and salt tolerance in *Oryza sativa*. Plant Molecular Biology. 1997; 33:857–865. 9106509 10.1023/a:1005702408601

[52] Miller G, Suzuki N, Ciftci-Yilmaz S, Mittler R. Reactive oxygen species homeostasis and signalling during drought and salinity stresses. Plant Cell Environment. 2010; 33: 453–467. 10.1111/j.1365-3040.2009.02041.x 19712065

[53] Arencibia AD, Carmona ER, Téllez P, Chan MT, Yu SM, Trujillo LE, et al An efficient protocol for sugarcane (*Saccharum spp*. L.) transformation mediated by *Agrobacterium tumefaciens*. Transgenic Research. 1998; 7(3): 213–222.

[54] Andrews S. FastQC: a quality control tool for high throughput sequence data. Babraham Bioinformatics. 2010; Available from: http://www.bioinformatics.babraham.ac.uk/projects/fastqc

[55] Bolger AM, Lohse M, Usadel B. Trimmomatic: A flexible trimmer for Illumina Sequence Data. Bioinformatics. 2014; 30: 2114–2120. doi: 10.1093/bioinformatics/btu170 24695404 10.1093/bioinformatics/btu170 PMC4103590

[56] Grabherr MG, Haas BJ, Yassour M, Levin JZ, Thompson DA, Amit I, et al Full-length transcriptome assembly from RNA-seq data without a reference genome. Nature Biotechnology. 2011; 29: 644–652. doi: 10.1038/nbt.1883 21572440 10.1038/nbt.1883 PMC3571712

[57] Haas BJ, Papanicolaou A, Yassour M, Grabherr M, Blood PD, Bowden J, et al De novo transcript sequence reconstruction from RNA-Seq: reference generation and analysis with Trinity. Nature Protocols. 2013; 8: 1–43 23845962 10.1038/nprot.2013.084 PMC3875132

[58] Zdobnov EM, Apweiler R. InterProScanm an integration platform for the signature-recognition methods in InterPro. Bioinformatics. 2001; 17: 847–848. 11590104 10.1093/bioinformatics/17.9.847

[59] Myhre S, Tveit H, Mollestad T, Lagreid A. Additional gene ontology structure for improved biological reasoning. Bioinformatics. 2006; 22: 2020–2027. doi: 10.1093/bioinformatics/btl334 16787968 10.1093/bioinformatics/btl334

[60] Finn RD, Coggill P, Eberhardt RY, Eddy SR, Mistry J, Mitchell AL, et al The Pfam protein families database: towards a more sustainable future. Nucleic Acids Research Database Issue. 2016; 44: D279–D285. 10.1093/nar/gkv1344 PMC4702930 26673716

[61] Marchler-Bauer A, Derbyshire MK, Gonzales NR, Lu S, Chitsaz F, Geer LY, et al CDD: NCBI's conserved domain database. Nucleic Acids Research 43(Database issue). 2015; D222–226. doi: 10.1093/nar/gku1221 25414356 10.1093/nar/gku1221 PMC4383992

[62] Marchler-Bauer A, Bryant SH. CD-Search: protein domain annotations on the fly. Nucleic Acids Research 32 (Web Server issue). 2004; W327–331. doi: 10.1093/nar/gkh454 15215404 10.1093/nar/gkh454 PMC441592

[63] Langmead B. Aligning short sequencing reads with Bowtie. Current Protocols in Bioinformatics. 2010; doi: 10.1002/0471250953.bi1107s32 21154709 10.1002/0471250953.bi1107s32 PMC3010897

[64] Robinson MD, McCarthy DJ, Smyth GK. edgeR: a Bioconductor package for differential expression analysis of digital gene expression data. Bioinformatics. 2010; 26: 139–140. doi: 10.1093/bioinformatics/btp616 19910308 10.1093/bioinformatics/btp616 PMC2796818

[65] R Development Core Team. R: A Language and environment for statistical computing. Vienna, Austria: The R Foundation for Statistical Computing; 2009 Available from: http://www.R-project.org/.

[66] Holm S. A simple sequentially rejective multiple test procedure. Scandinavian Journal of Statistics. 1979; 6: 65–70.

[67] Wickham H. ggplot2: Elegant Graphics for Data Analysis. Springer-Verlag New York, 2009 Available from: https://cran.r-project.org/web/packages/ggplot2/index.html

[68] Ling H, Wu Q, Guo J, Xu L, Que Y. Comprehensive Selection of Reference Genes for Gene Expression Normalization in Sugarcane by Real Time Quantitative RT-PCR. PLoS ONE. 2014; https://doi.org/10.1371/journal.pone.0097469 10.1371/journal.pone.0097469 PMC4019594 24823940

[69] Barrs HD, Weatherley PE. A re-examination of the relative turgidity technique for estimating water deficits in leaves. Australian Journal of Biological Sciences. 1962; 24: 519–570.

[70] Carillo P, Mastrolonardo G, Nacca F, Parisi D, Verlotta A, Fuggi A. Nitrogen metabolism in durum wheat under salinity: accumulation of proline and glycine betaine. Functional Plant Biology. 2008; 35: 412–426. 10.1071/FP08108 32688798

