# Supplementary material for: Transcriptional profiling of sugarcane leaves and roots under progressive osmotic stress reveals a regulated coordination of gene expression in a spatiotemporal manner
Source: PLoS One. 2017 Dec 11;12(12):e0189271. doi: 10.1371/journal.pone.0189271 (PMC5724895; doi:10.1371/journal.pone.0189271)
Supplement: S1 Table — (PDF) [file pone.0189271.s001.pdf]

Table S1. List of sugarcane genes and primers used for the RT-qPCR validation.

GENE	PRIMER NAME	PRIMER SEQUENCE	Tm	AMPLICON SIZE
NAC transcription factor 25-like (*NAC025*)	qNAC25-Fw	5’-CAGCAGAGCCCACTCAAGA-3’	60°C	151 bp
	qNAC25-Rev	5’-CAAACACGGAGAAGGCGTTG-3’		
Bidirectional sugar transporter SWEET6a-like (*SWEET6a*)	qSWT6a-Fw	5’-CATCTTCTTCATCTACTCCGAC-3’	60°C	77 bp
	qSWT6a-Rev	5’-CCACGAACAGAATCTCCAC-3’		
abscisic acid 8 -hydroxylase 3 -like (*ABIP3*)	qABIP3-FW	5’-ATACCATGAAGACGCTCACCTTC-3’	64°C	111 bp
	qABIP3-REV	5’-CTTTCTCCACGATCAAGTAGT-3’		
Phosphatidylinositol 4,5 -bisphosphate (*PIP2*)	qPIP2-FW	5’-GTGTTCATGGTGCACCTGG-3’	65°C	177 bp
	qPIP2-Rev	5’-GAGGATGTACTGGTGGTAGAAGG-3’		
