# Supplementary material for: Transcriptional profiling of sugarcane leaves and roots under progressive osmotic stress reveals a regulated coordination of gene expression in a spatiotemporal manner
Source: PLoS One. 2017 Dec 11;12(12):e0189271. doi: 10.1371/journal.pone.0189271 (PMC5724895; doi:10.1371/journal.pone.0189271)
Supplement: S1 Fig — (PDF) [file pone.0189271.s002.pdf]

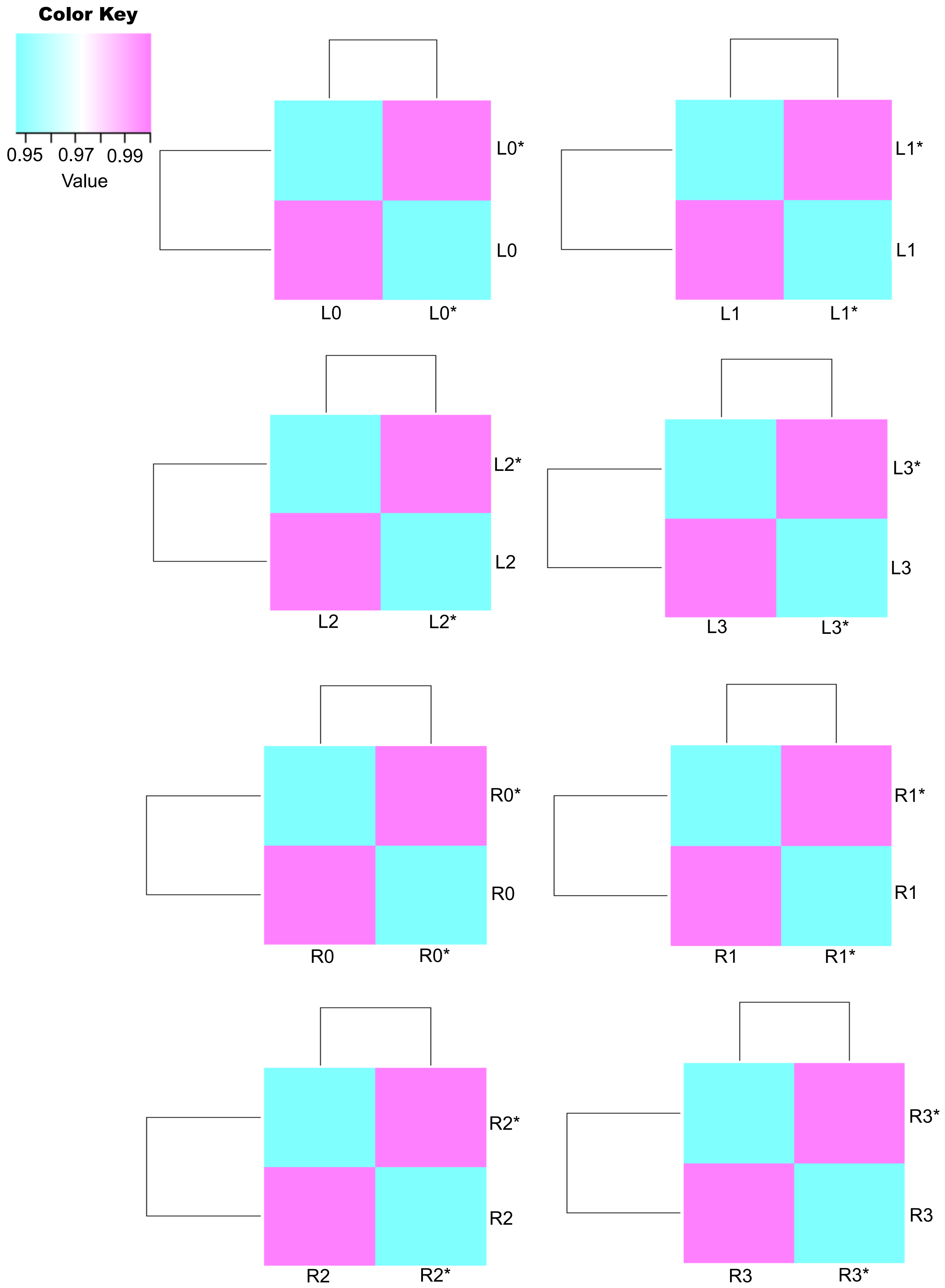

Figure S1. Pearson's correlation analysis among replicates of each biological sample. L=leaf, R=root, L0 and R0= non-stress treatment, L1 and R1=24 h treatment ASI, L2 and R2=48 h treatment ASI, L3 and R3=72 h treatment ASI. *=biological replicate.
