# Supplementary material for: Transcriptional profiling of sugarcane leaves and roots under progressive osmotic stress reveals a regulated coordination of gene expression in a spatiotemporal manner
Source: PLoS One. 2017 Dec 11;12(12):e0189271. doi: 10.1371/journal.pone.0189271 (PMC5724895; doi:10.1371/journal.pone.0189271)
Supplement: S2 Fig — (PDF) [file pone.0189271.s003.pdf]

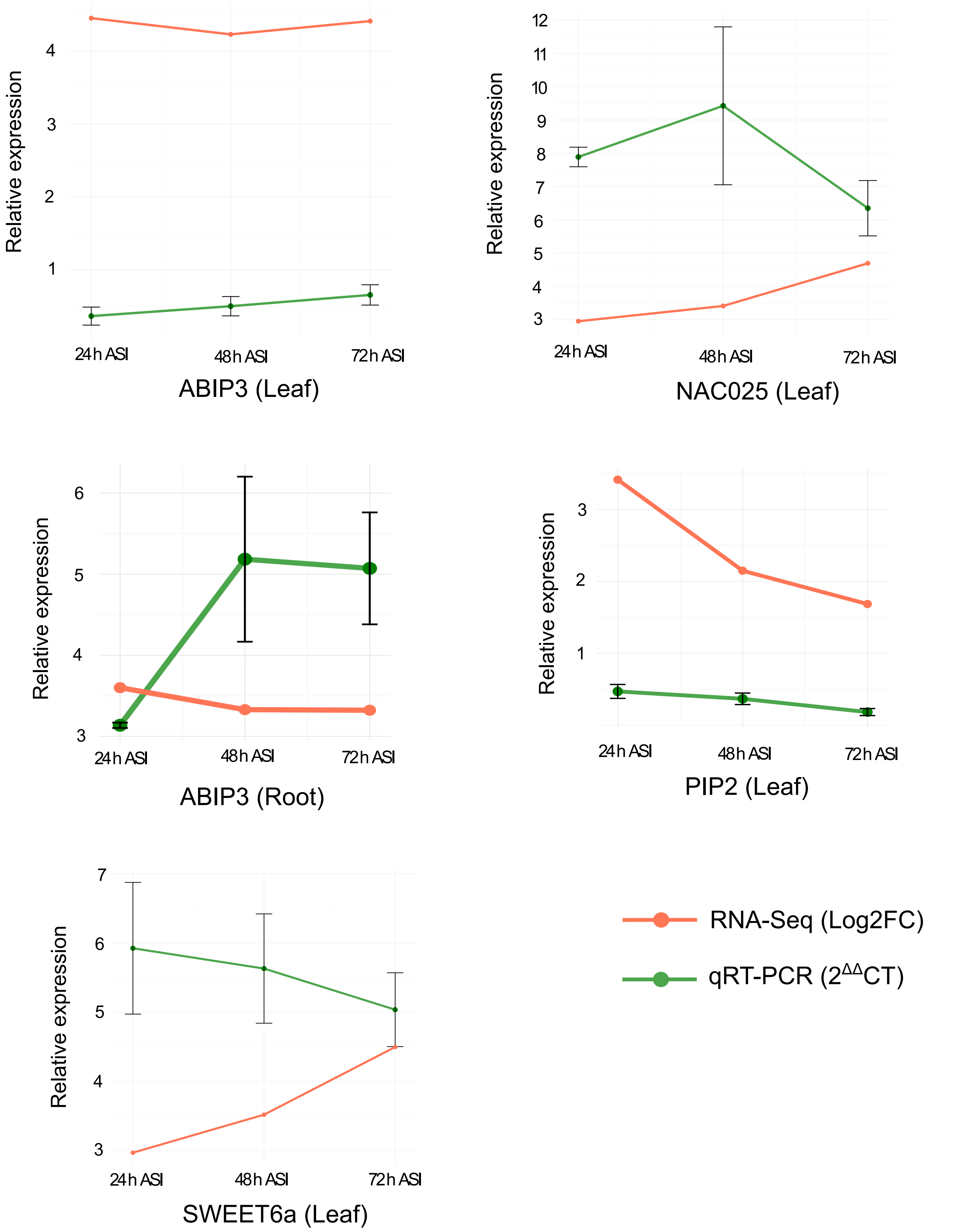

Figure S2. qRT-PCR analysis of five significatively regulated genes randomly selected into the osmotic stress treatments. The x-axis represent the three times of stress tretments, y-axis represent the relative gene expression levels. Error bars were obtained from three technical replicates.
