# Supplementary material for: Transcriptional profiling of sugarcane leaves and roots under progressive osmotic stress reveals a regulated coordination of gene expression in a spatiotemporal manner
Source: PLoS One. 2017 Dec 11;12(12):e0189271. doi: 10.1371/journal.pone.0189271 (PMC5724895; doi:10.1371/journal.pone.0189271)
Supplement: S3 File — (PDF) [file pone.0189271.s006.pdf]

Permission is granted to PLoS ONE to publish under the Creative Commons Attributions License (CCAL), CC BY 4.0 open access license the following KEGG pathway map image in the article "Transcriptional profiling of sugarcane leaves and roots under progressive osmotic stress reveals a regulated coordination of gene expression in a spatiotemporal manner" written by Luis C. Rodriguez Zapata and colleagues:

- Starch and sucrose metabolism (map00500)

Sincerely,
Miwako Matsumoto
Kanehisa Laboratories

KEGG reference:

Kanehisa M, Furumichi M, Tanabe M, Sato Y, Morishima K. KEGG: new perspectives on genomes, pathways, diseases and drugs. Nucleic Acids Research. 2017; 45:D353-D361.

Links:

http://creativecommons.org/licenses/by/4.0/
