# Supplementary material for: Transcriptional profiling of sugarcane leaves and roots under progressive osmotic stress reveals a regulated coordination of gene expression in a spatiotemporal manner
Source: PLoS One. 2017 Dec 11;12(12):e0189271. doi: 10.1371/journal.pone.0189271 (PMC5724895; doi:10.1371/journal.pone.0189271)
Supplement: S4 File — (PDF) [file pone.0189271.s007.pdf]

S4 File. Abbreviations and acronyms

1-SST: Sucrose:sucrose 1-fructosyltransferase 1; ABA: abscisic acid; ACC: 1-Aminocyclopropane-1-carboxylic acid; bZIP: Basic leucine zipper; CAT1: Cationic amino acid transporter 1; CathB3: Cathepsin B-like 3; CESA4: Cellulose synthase A catalytic subunit 4 [UDP-forming]; CESA5: Cellulose synthase 5; CHI: Chalcone-flavonone isomerase; Chl a/b-binding (LCHII)1: Chlorophyll a-b binding of LHCII type 1; CHLG: Chlorophyll synthase, chloroplastic; CHLD: Magnesium-chelatase subunit, chloroplastic; CHS: Chalcone synthase 1; CPRF2: Light-inducible protein; CSLD2: Cellulose synthase D2; CuZn-SOD: Superoxide dismutase [Cu-Zn]1; DEGs: Differentially expressed genes DES2: Fatty acid desaturase 2; DIN1: Senescence-associated DIN1; DREB: Dehydration-responsive element-binding; E2FB: Transcription factor E2FB; EMB1444: Protein EMBRYO DEFECTIVE 1444, Transcription Factor; ERF: Ethylene-responsive transcription factor; G6PDH6: Glucose-6-phosphate 1-dehydrogenase, cytoplasmic isoform; GA2ox8: Gibberellin 2-beta-dioxygenase 8; GAPCP1: Glyceraldehyde-3-phosphate dehydrogenase 1, chloroplastic; GO: Gene ontology; GR: Glutathione reductase, cytosolic; H^+-ATPase: Plasma membrane ATPase 1; HCF136: Photosystem II stability assembly factor HCF136, chloroplastic; HOX22: Homeobox-leucine zipper; hs32: Phosphosulfolactate synthase-related; KEGG: Kyoto encyclopedia of genes and genomes; LEA: Late embryogenesis abundant Mn-SOD: Superoxide dismutase [Mn], mitochondrial-like; MYB(APL): MYB family transcription factor APL-like; NAA: Naphthaleneacetic acid; NAC: NAM, ATAF, CUC1/2; NGS: New generation sequencing; NYC1: Probable chlorophyll(ide) b reductase NYC1, chloroplastic; P5CS: Delta-1-pyrroline-5-carboxylate synthase; PAC: PALE CRESS protein, chloroplastic; PAL: Phenylalanine ammonia-lyase; PEG: polyethylene glycol; PEP1: Phosphoenolpyruvate carboxylase 1; PHOT1A: Phototropin-1A; PIP: Plasma membrane intrinsic protein; PLDGAMMA1: Phospholipase D gamma 1; PLT5: Polyol transporter 5; PNP1: Probable polyribonucleotide nucleotidyltransferase 1, chloroplastic; PP2C: Probable phosphatase 2C; PPCK2: Phosphoenolpyruvate carboxylase kinase2; QUA2: Probable pectin methyltransferase; RF2b: Transcription factor RF2b; RFS: Galactinol-sucrose galactosyltransferase; RHD3: ROOT HAIR DEFECTIVE 3; SGRL: STAY-GREEN, chloroplastic-like; SSII-2: Soluble starch synthase 2-2, chloroplastic amyloplastic; SUS1: Sucrose synthase 1; TCTP: Translationally-controlled tumor homolog; TDT: Tonoplast dicarboxylate transporter; TIP: Tonoplast intrinsic protein; TRX1:Thioredoxin H1; TXNRD2: Thioredoxin reductase 2; ZEP: Zeaxanthin epoxidase, chloroplastic-like.
