# Supplementary figures and images for: Transcriptional profiling of sugarcane leaves and roots under progressive osmotic stress reveals a regulated coordination of gene expression in a spatiotemporal manner
Source: PLoS One. 2017 Dec 11;12(12):e0189271. doi: 10.1371/journal.pone.0189271 (PMC5724895; doi:10.1371/journal.pone.0189271)

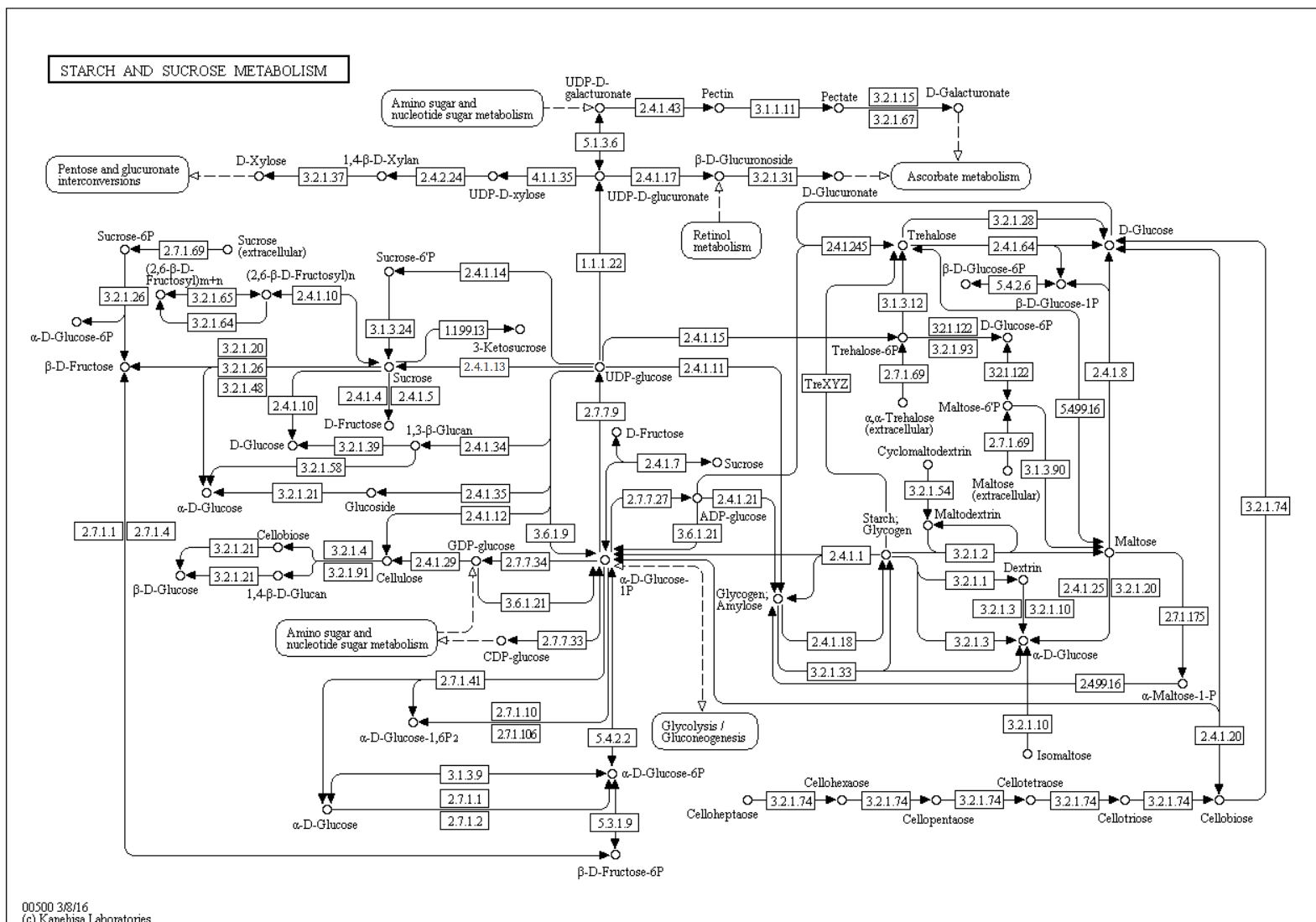

Supplement: S5 File — (PNG) [file pone.0189271.s008.png]
